# Paired evaluation of machine-learning models characterizes effects of confounders and outliers

**DOI:** 10.1016/j.patter.2023.100824

**Published:** 2023-08-11

**Authors:** Maulik K. Nariya, Caitlin E. Mills, Peter K. Sorger, Artem Sokolov

(Patterns *4*, 100791; August 11, 2023)

In Figure 4B of the originally published article, the erroneous AUC values 0.82 and 0.88 were mistakenly included. The values 0.82 and 0.88 should be 0.10 and 0.80, respectively. The error has now been corrected online, and the authors regret any confusion that it has caused.

**Figure 4B:**
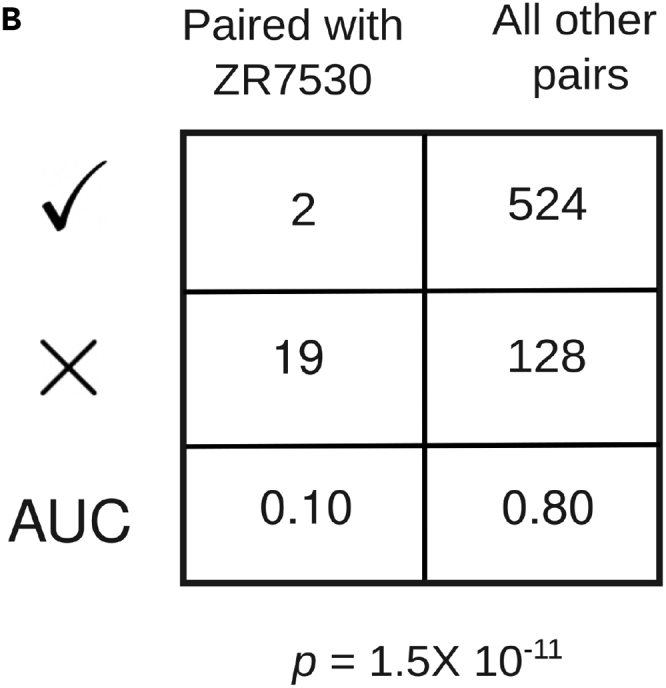
Paired evaluation detects outlier cell lines in the context of sensitivity to Torin2 (corrected)

**Figure 4B:**
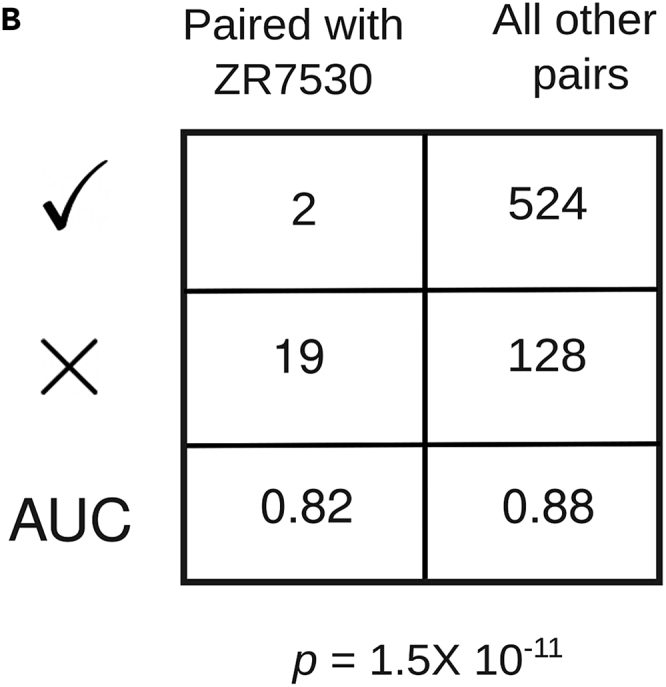
Paired evaluation detects outlier cell lines in the context of sensitivity to Torin2 (original)

