# Supplementary material for: Paired evaluation of machine-learning models characterizes effects of confounders and outliers
Source: Patterns (N Y). 2023 Jul 7;4(8):100791. doi: 10.1016/j.patter.2023.100791 (PMC10435952; doi:10.1016/j.patter.2023.100791)
Supplement: Document S1. Figures S1–S9 and Table S1 [file mmc1.pdf]

Patterns, Volume *4*

Supplemental information

Paired evaluation of machine-learning models characterizes effects of confounders and outliers

Maulik K. Nariya, Caitlin E. Mills, Peter K. Sorger, and Artem Sokolov

Supplementary information

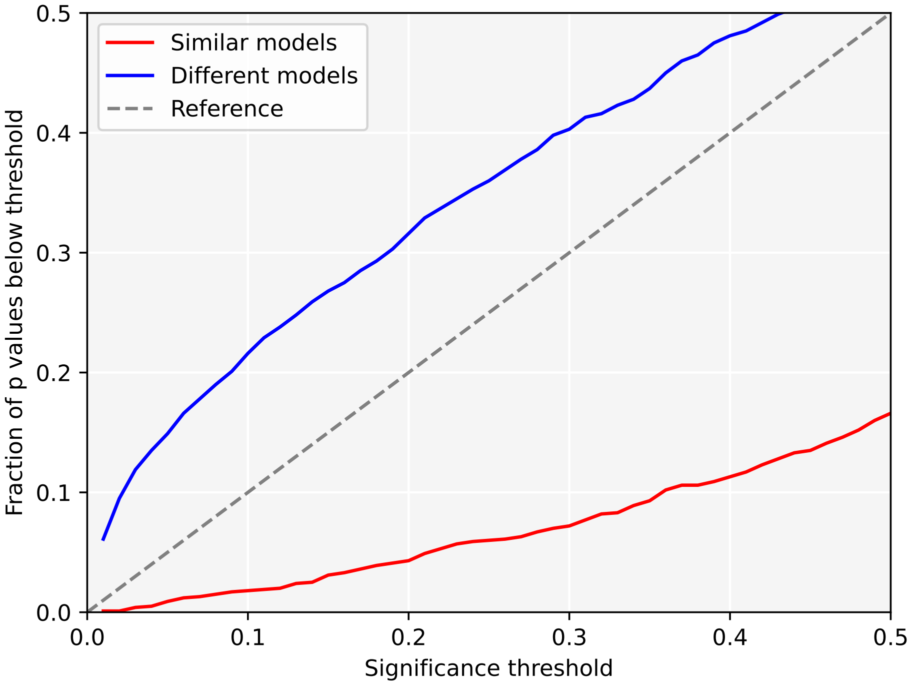

Figure S1. The probability of paired evaluation rejecting the null hypothesis that two models have the same performance. Shown is the fraction of p values from 1,000 two-sided Fisher's Exact tests that fall below a given significance threshold. Each p value was derived on a random 80/20 train/test split of the breast cancer dataset (see Results) by comparing the performance of two random forest models trained with identical hyperparameter values (red) or one random forest model and one linear regression model (blue). The expected "no information" rate is presented as a dashed line for reference.

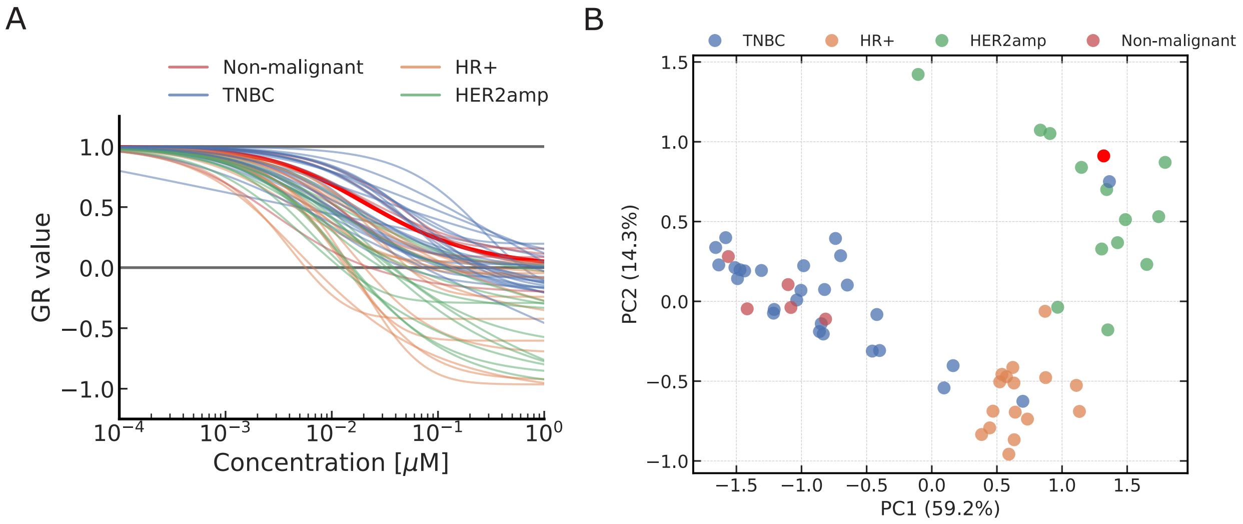

Figure S2. The location of ZR7530 data relative to other cell lines. A.) Growth rates curves for torin2 across all breast cancer cell lines. **B.)** Principal components analysis of the baseline RNA-seq data, computed in the space of the top 20 most important genes (Figure 3). The "outlier" cell line ZR7530 is highlighted in red, all other cell lines are colored by their subtypes.

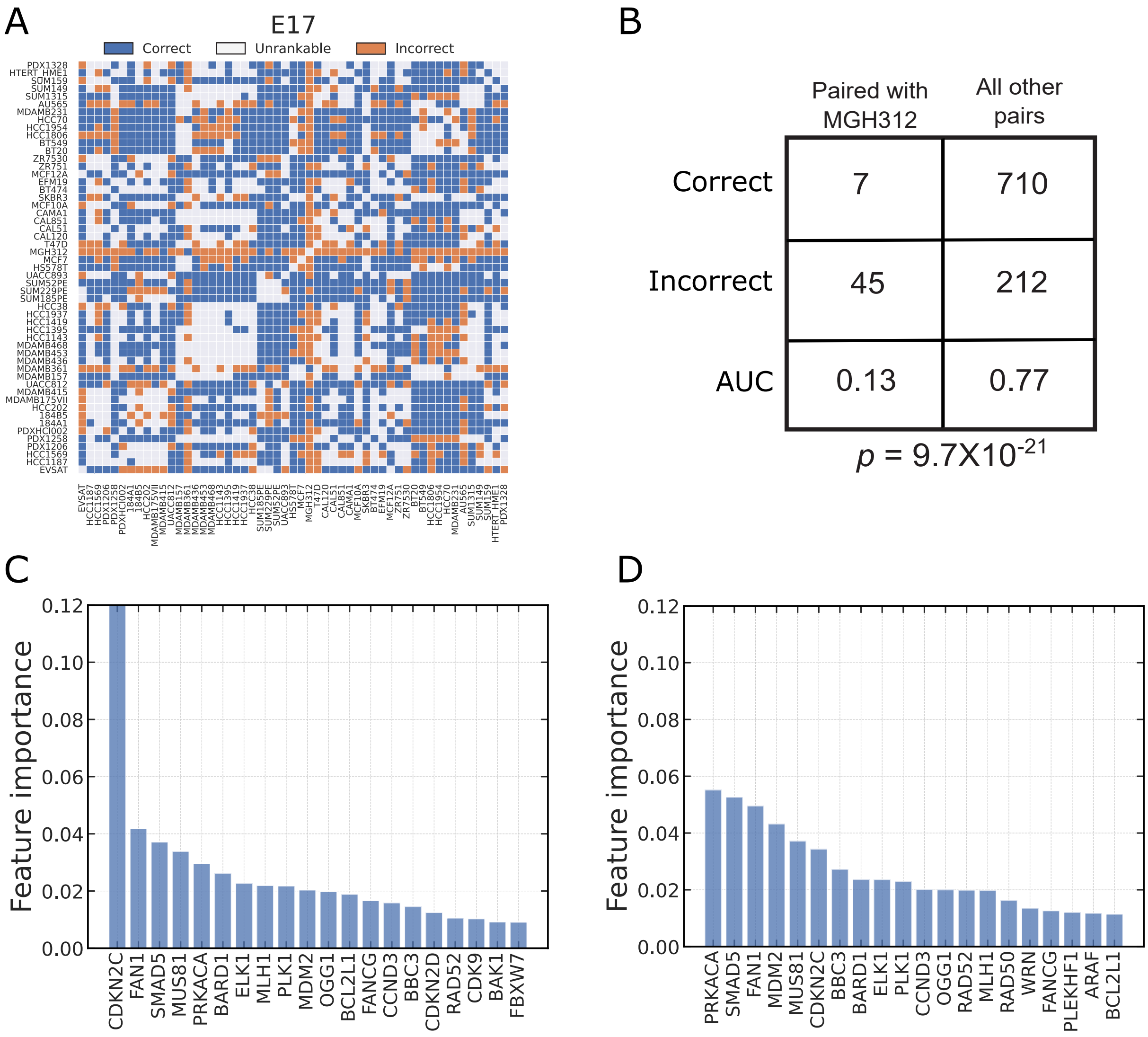

Figure S3. Outlier detection for E17. The interpretation of all panels is analogous to Figure 3.

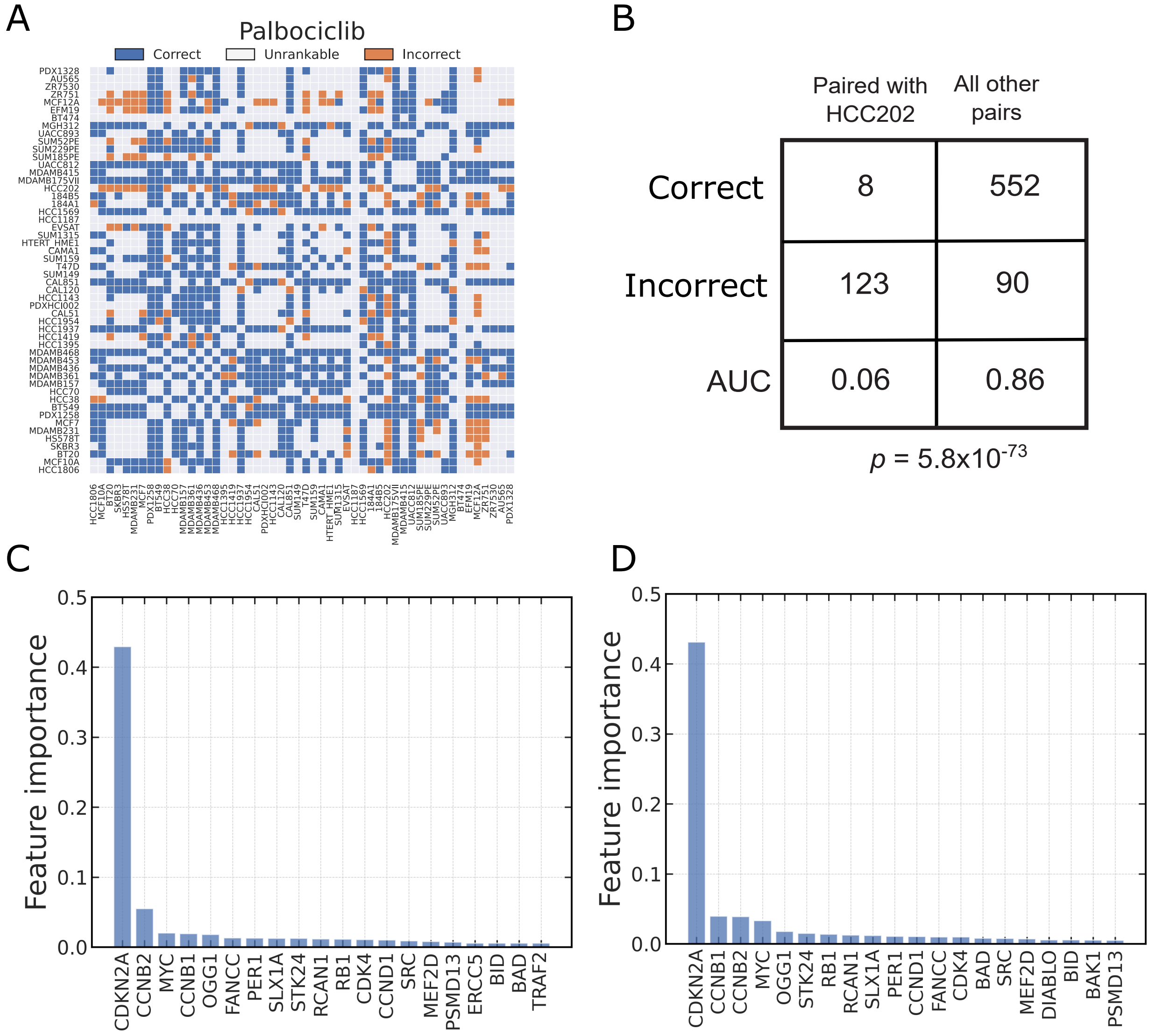

Figure S4. Outlier detection for palbociclib. The interpretation of all panels is analogous to Figure 3.

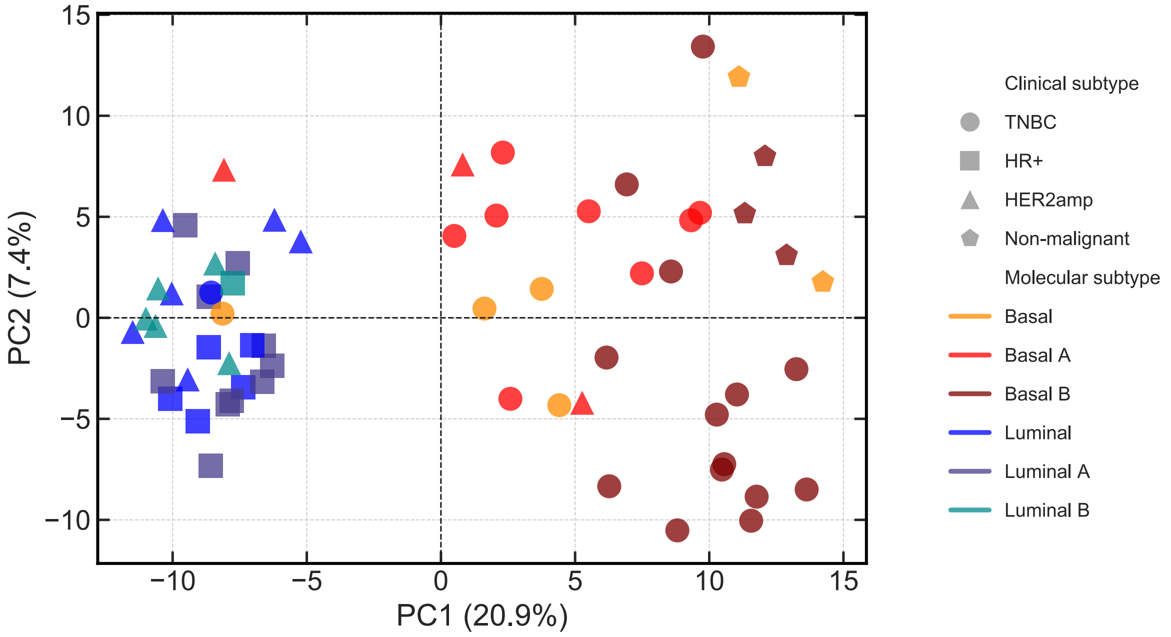

Figure S5. Principal components analysis of baseline RNAseq expression. The points represent individual breast cancer cell lines, shaped according to clinical subtype and colored by molecular subtype.

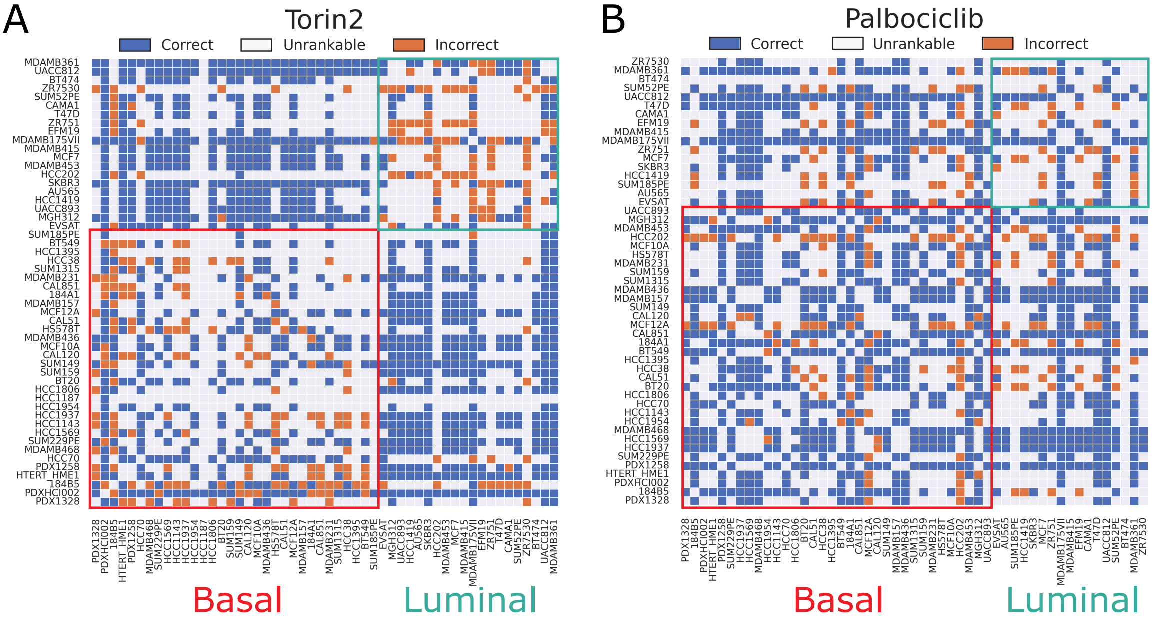

Figure S6. Effect of subtype in model prediction for torin2 and palbociclib.

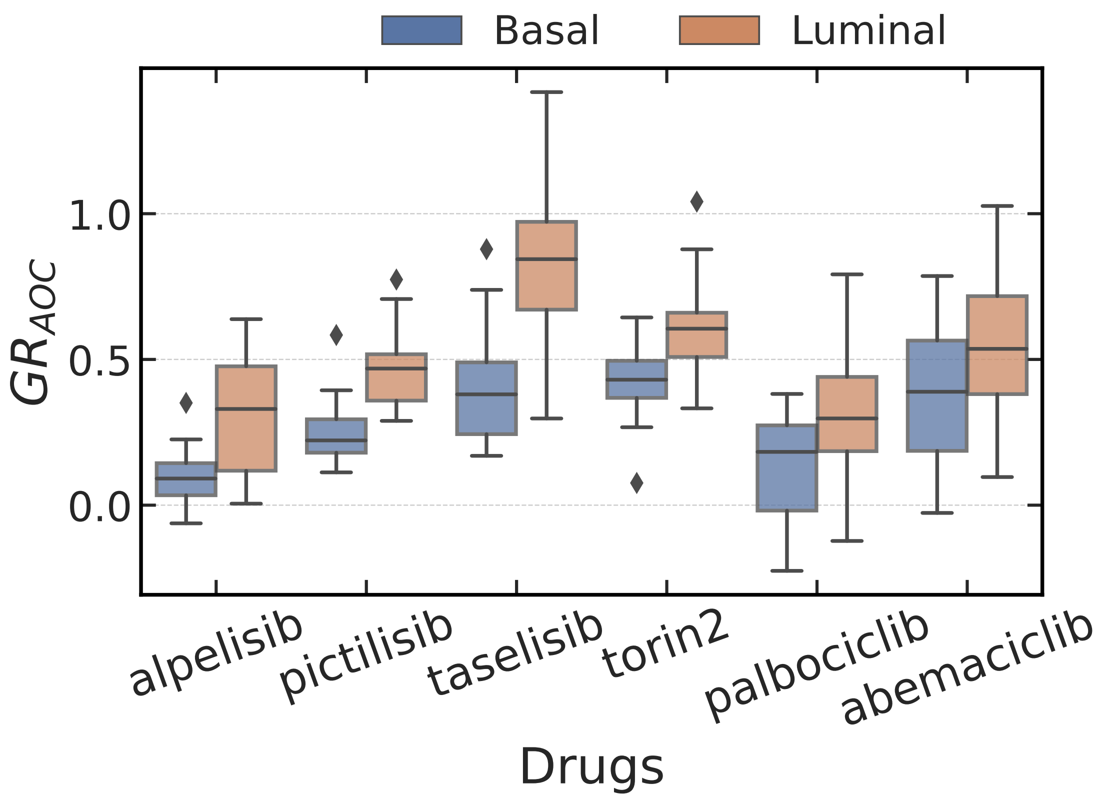

Figure S7. The distribution of GR_{AOC} for selected drugs. The values are plotted separately for basal (blue) and luminal (orange) cell lines.

Moderate vs mild

Mild-centric

	AOD confounded	AOD matched
✓	59	46
✗	29	42
AUC	0.67	0.52

$p = 0.03$

Moderate-centric

	AOD confounded	AOD matched
✓	133	138
✗	53	48
AUC	0.71	0.74

$p = 0.76$

Moderate vs severe

Moderate-centric

	AOD confounded	AOD matched
✓	162	168
✗	40	34
AUC	0.80	0.83

$p = 0.81$

Severe-centric

	AOD confounded	AOD matched
✓	51	60
✗	12	3
AUC	0.81	0.95

$p = 1.0$

Figure S8. 2x2 contingency tables showing the performance of logistic regression models trained to distinguish between mild, moderate and severe stages of Alzheimer's Disease in the presence of Age of Death (AOD) as a confounder.

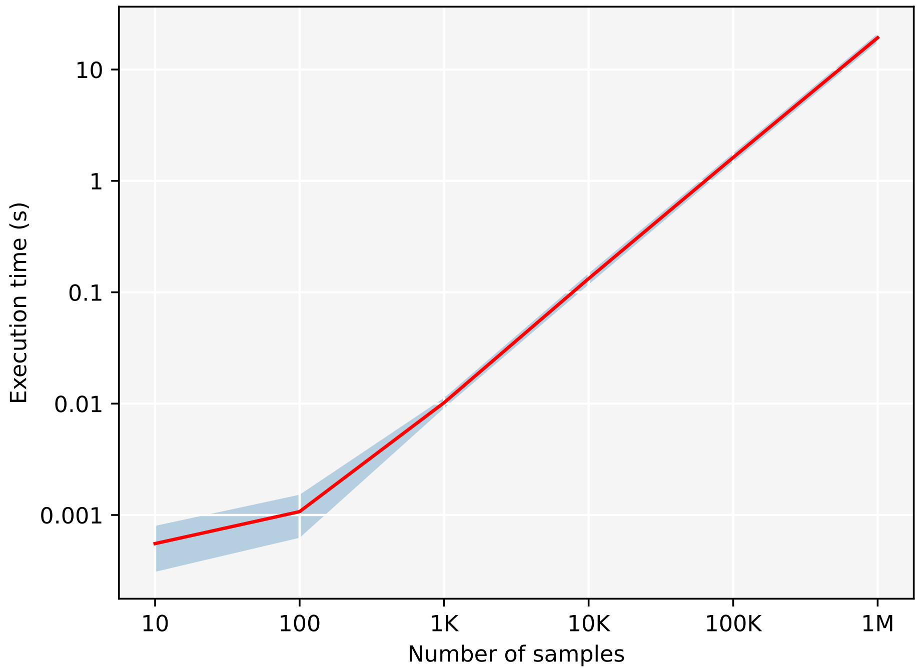

Figure S9. Execution time of the $O(n \log n)$ paired evaluation implementation as a function of dataset size. Shown are statistics collected over 30 runs of the method on randomly-generated sets of scores and labels. The shaded areas (blue) are one standard deviation away from the mean (red). All measurements were made in a standard Gitpod execution environment (4 vCPU, 8GB RAM) using the Python time() function.

Table S1: The total number of rankable pairs used to evaluate predictors of drug sensitivity from mRNA expression.

Agent	Number of pairs		Agent	Number of pairs
Paclitaxel	443		BSJ-01-175	616
Doxorubicin	469		BSJ-03-123	304
Taselisib_GDC0032	714		BSJ-03-124	435
Pictilisib_GDC0941	358		BVD523	138
Torin2	389		CFI-400945	553
Vorinostat	112		E17	702
Ipatasertib_GDC0068	333		FMF-03-145-1	705
Everolimus	535		FMF-03-146-1	42
Tivantinib_ARQ197	125		FMF-04-107-2	787
Cabozantinib	66		FMF-04-112-1	7
Saracatinib_AZD0530	296		Flavopiridol	99
Dasatinib	570		GSK2334470	366
Palbociclib_PD0332991	428		LEE011_Ribociclib	524
Dinaciclib_SCH727965	224		LY2606368	747
AZD7762	592		LY3023414	721
Olaparib_AZD2281	50		MFH-2-90	837
Alpelisib_BYL719	367		Pin1-3	50
A-1210477	7		R0-3306	125
Buparlisib_NVP-BKM120	188		Rucaparib	198
INK128_MLN0128	526		SHP099	61
PF-4708671	65		SY-1365	735
Neratinib_HKI272	588		THZ-P1-2	356
Cediranib_AZD2171	220		THZ-P1-2R	37
Ceritinib_LDK378	205		THZ1	399
Trametinib_GSK1120212	460		THZ531	352
Luminespib_NVP-AUY922	322		YKL-5-124	570
Abemaciclib_LY2835219	559		ZZ1-33B	852
Volasertib_BI6727	363		senexin b	150
ABT-737	131			
TGX221	190			
AZD1775	668			
AZD2014	693			
AZD5363	531			
AZD6738	294			
BJP-6-5-3	0			
BMS-265246	689			
