# Supplementary material for: Paired evaluation of machine-learning models characterizes effects of confounders and outliers
Source: Patterns (N Y). 2023 Jul 7;4(8):100791. doi: 10.1016/j.patter.2023.100791 (PMC10435952; doi:10.1016/j.patter.2023.100791)
Supplement: Document S2. Article plus supplemental information [file mmc2.pdf]

Article

Patterns

# Paired evaluation of machine-learning models characterizes effects of confounders and outliers

## Highlights

- Paired evaluation enables detection of outliers and statistical comparison of models
- The method can evaluate the impact of confounders without model retraining
- The method scales efficiently to millions of data points

## Authors

Maulik K. Nariya, Caitlin E. Mills, Peter K. Sorger, Artem Sokolov

## Correspondence

artem_sokolov@hms.harvard.edu

## In brief

A simple method based on pairing of data samples allows for effective evaluation of performance in machine-learning models. The method helps reveal how outliers and confounders affect performance estimates and model interpretation. An effective implementation of the method enables its application in modern large-scale datasets.

Nariya et al., 2023, Patterns *4*, 100791
August 11, 2023 © 2023 The Author(s).
https://doi.org/10.1016/j.patter.2023.100791

CellPress

Patterns

CellPress
OPEN ACCESS

Article

# Paired evaluation of machine-learning models characterizes effects of confounders and outliers

Maulik K. Nariya,[1,2] Caitlin E. Mills,[1] Peter K. Sorger,[1,2] and Artem Sokolov[1,3,4,*]
[1]Laboratory of Systems Pharmacology, Harvard Program in Therapeutic Science, Harvard Medical School, Boston, MA 02115, USA
[2]Department of Systems Biology, Harvard Medical School, Boston, MA 02115, USA
[3]Department of Biomedical Informatics, Harvard Medical School, Boston, MA 02115, USA
[4]Lead contact
*Correspondence: artem_sokolov@hms.harvard.edu
https://doi.org/10.1016/j.patter.2023.100791

**THE BIGGER PICTURE** Limited data availability, outliers, and confounding variables are ubiquitous in biological and clinical datasets and present challenges for evaluation and comparison of machine-learning models. We present a method that uses simple pairing of data samples to overcome those challenges and reveal how individual outliers and confounders impact performance estimates without the need to train multiple models. We also propose an efficient implementation of the method, allowing it to be applied at scale.

1 2 3 4 5 **Development/Pre-production:** Data science output has been rolled out/validated across multiple domains/problems

## SUMMARY

The true accuracy of a machine-learning model is a population-level statistic that cannot be observed directly. In practice, predictor performance is estimated against one or more test datasets, and the accuracy of this estimate strongly depends on how well the test sets represent all possible unseen datasets. Here we describe paired evaluation as a simple, robust approach for evaluating performance of machine-learning models in small-sample biological and clinical studies. We use the method to evaluate predictors of drug response in breast cancer cell lines and of disease severity in patients with Alzheimer's disease, demonstrating that the choice of test data can cause estimates of performance to vary by as much as 20%. We show that paired evaluation makes it possible to identify outliers, improve the accuracy of performance estimates in the presence of known confounders, and assign statistical significance when comparing machine-learning models.

## INTRODUCTION

Effectively evaluating the performance of predictive computational models is a crucial aspect of machine learning. Knowing when a model is accurate allows for reliable predictions on new data and provides valuable insights about which features in the training data carry predictive information. However, the true accuracy of a model is a population-level statistic that is generally unknown, because it is impossible to consider all—potentially infinitely many—datasets to which a model will be applied. Model performance must therefore be estimated by appropriately sampling available data, and reliable estimates require a sufficient number of points to be adequately representative of the population. The presence of systematic biases and confounding variables can lead to incorrect accuracy estimates and inflated confidence in machine-learning models that are subsequently found to perform poorly in deployment.[1] This is closely related to the well-known issue of overfitting,[2] whereby a model trained on one set of data points fails to generalize to a new set of data. Conventional methods for performance evaluation can fail to detect overfitting when the same biases are present in training and test data. Robust performance estimates must therefore detect and account for these biases to accurately represent how the model would behave in the larger space of all possible data points.

When data are limited (as they commonly are in biomedicine), model performance is routinely evaluated using cross-validation, which involves withholding a portion of the available data and using the remainder to train a model, which is then evaluated against the withheld portion.[3,4] Widely used variants of


This is an open access article under the CC BY license (http://creativecommons.org/licenses/by/4.0/).

cross-validation include *k*-fold; leave-one-out; Monte Carlo methods, in which a fixed proportion of data are repeatedly sampled and withheld for evaluation; and bootstrap methods, where the withheld portion is automatically defined by the data not sampled for training.[5] In their standard formulations, none of these methods explicitly account for the presence of systematic biases and confounders in the data, and model accuracy estimates obtained by these methods may not always reflect true predictor performance, particularly when dataset sizes are small.

The limitation of data availability is particularly prominent in -omics datasets, which commonly contain many molecular measurements (ca. $10^4$ for genome-scale data) from a relatively small number of samples (10–100). While conducting more experiments to increase sample size is sometimes possible, the small-sample issue is insurmountable in other cases due to the limited availability of biological material (number of available patient specimens, for example) and the significant cost associated with molecular profiling. For example, cell culture studies focused on breast cancer are generally limited to the $\sim$75 commercially available breast cancer cell lines. While deriving new cell lines is possible, it is time consuming and expensive.[6] Moreover, new lines potentially suffer from the same limitations as existing lines with respect to confounders. The discrepancy between the low number of samples and the large number of molecular features available for any one sample introduces low-signal scenarios. For example, the availability of deep gene expression data that cover thousands of expressed genes in a small number of samples makes it difficult to detect relevant transcriptional changes in the overall expression variance.[7] A low number of samples can also lead to stratification bias, because it is not always possible to partition a small but discrete number of data instances into cross-validation folds in a way that preserves the statistical properties of the entire dataset in each fold.[8] Together, these issues represent a substantial challenge in making accurate estimates of performance for models trained on -omics and similar datasets.

In addition to challenges arising when sample number is low, biological and clinical datasets often contain both known and unknown confounding relationships among variables. For example, a recent study found that the dominant signal in a prototypical large multi-center drug-response screen aligned with the location at which the data were collected and not the drug or cell line.[9] Knowing when a machine-learning model inadvertently learns to recognize such a lurking variable can help prevent spurious correlations and erroneous conclusions. A popular approach for dealing with confounding and lurking variables is to modify the input data in a way that removes or reduces their effect, as implemented by ComBat,[10] surrogate variable analysis,[7] removal of unwanted variation,[11] and linear models for microarray data.[12] However, modification of the original data can inadvertently introduce new artifacts that erroneously amplify differences between data groups and inflate estimates of model performance.[13] Some batch-correcting methods also assume an underlying statistical distribution for the data, making them inappropriate for scenarios in which the data distribution is unknown.

In this work, we use paired evaluation to systematically examine how a predictive model scores pairs of test data samples to generate a detailed decomposition of performance estimates. The evaluation method was originally proposed in the context of regression problems,[14] but its full potential remained unexplored. Here, we generalize paired evaluation to all machine-learning tasks and demonstrate how the method can identify potential data outliers, assign statistical significance when comparing machine-learning methods, and serve as a non-parametric method to accurately estimate model performance in the presence of known confounders without requiring modification of the underlying data. We consider two small-sample prediction tasks that leverage real-world datasets with known confounders: prediction of drug sensitivity in breast cancer cell lines, which is confounded by subtype (clinical, i.e., hormone-receptor positive [HR+], HER2 amplified, triple negative; and molecular, i.e., luminal, basal); and prediction of Alzheimer's disease (AD) severity in postmortem brain specimens, which is confounded by an individual's chronological age. We show that minor variations in how the test data are paired for evaluation can reveal significant effects hidden by traditional approaches to model evaluation, and that the exclusion of outliers detected by paired evaluation can affect model interpretation. Last, we show that paired evaluation can be implemented efficiently using the "inversion counting" algorithm, which enables its application to large-scale datasets, such as those comprising single-cell RNA sequencing (RNA-seq) measurements.

## RESULTS

Throughout this work, we quantify model accuracy using a popular metric, the area under the receiver operating characteristic curve (AUC).[15] In binary classification, the AUC can be interpreted as the probability that a randomly chosen positive sample is correctly ranked above a randomly chosen negative sample.[16] This interpretation integrates naturally with paired evaluation, which considers one pair of samples at a time and evaluates a predictor based on whether it ranks that pair correctly. We define a pair of samples to be rankable if their labels can be ordered—given experimental error and other uncertainty—by the corresponding data representation (e.g., the temporal arrangement of events [disease progression or death] in a survival dataset). The fraction of pairs ranked correctly is a direct estimate of AUC (Figure 1A). Paired evaluation is agnostic to the underlying machine-learning method and can be applied in any cross-validation setting that allows for pooling and comparison of predictions from multiple test folds. A special case of this is leave-pair-out cross-validation (LPOCV), in which a separate model is trained for each test pair.[17] LPOCV is particularly relevant for small-sample datasets with low signal-to-noise ratios, because it has been shown to be less susceptible to stratification bias than other popular cross-validation schemes.[16–18]

Paired evaluation is not limited to regression (where the method was originally proposed[14]) or binary classification and can be applied to any machine-learning task that allows for an ordering of sample labels, including information retrieval, recommender systems, and survival analysis. To account for instrument error and other sources of uncertainty in -omics datasets, we introduce an optional meta-parameter, $\delta$, which sets a minimum required distance of separation in the label space for a

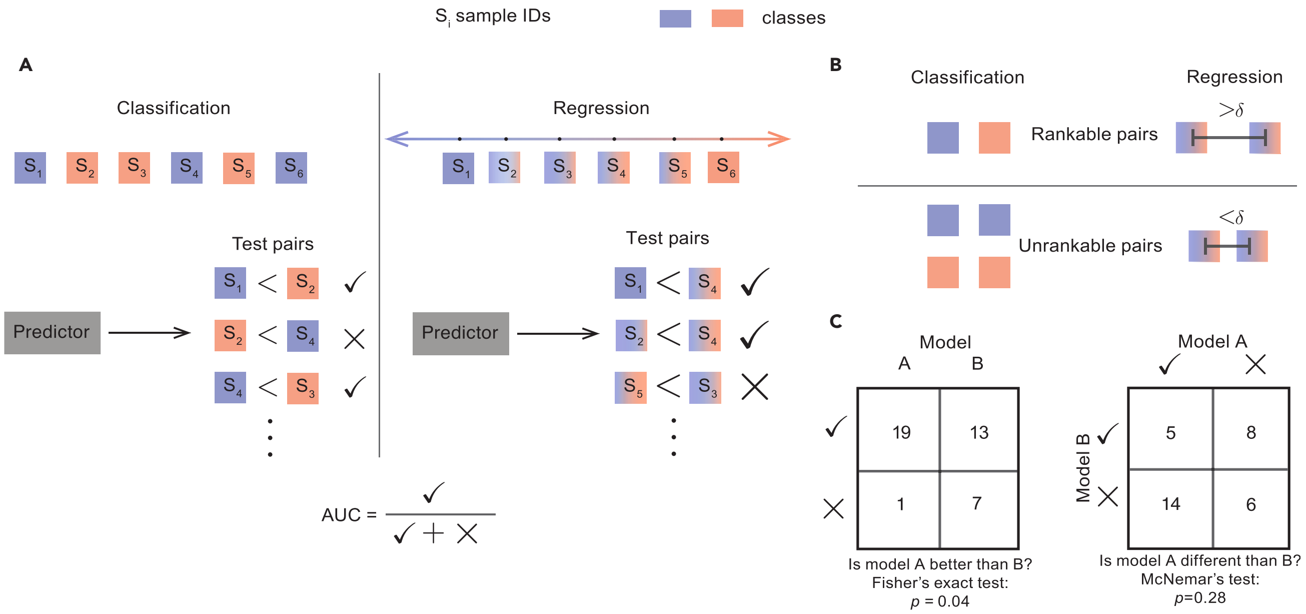


**Figure 1. A schematic representation of paired evaluation**

(A) Individual samples in a test dataset are represented by squares, colored according to their true labels in binary classification and linear regression settings. The test dataset is broken up into rankable pairs, and a predictor is asked to score each pair separately. The scores are used to determine whether a given pair was ranked correctly (✓) or incorrectly (X), and the AUC is determined by the fraction of correctly ranked pairs.

(B) The criteria for a valid rankable test pair. In binary classification, two samples are considered rankable if they belong to the opposite classes; in linear regression, a rankable pair of samples requires that the difference between their labels is greater than a predefined meta-parameter $\delta$.

(C) An example comparison of two models (A and B). A 2 × 2 contingency table tallies the correctly and incorrectly ranked pairs by each model. Statistical significance of the difference in method performance is assessed by Fisher's exact test and McNemar's test.

See also Figure S1.

pair of samples to be considered rankable (Figure 1B). In all settings, AUC is estimated as the fraction of rankable pairs that are ranked correctly by a model.

The primary advantage of paired evaluation is that it allows models A and B to be compared against each other based on their ability to correctly rank individual pairs of datapoints. This is both more informative than the AUC value alone and more detailed than the standard evaluation measurements produced by the popular k-fold and leave-one-out cross-validation methods, allowing for a deeper characterization of model performance in small-sample studies. When comparing two models in a paired evaluation setting, statistical significance can be assessed by simple construction of a two-by-two contingency table and application of standard statistical tests,[19] such the Fisher's exact test (Figure 1C). Other tests, such as McNemar's, make it possible to detect instances in which two models perform differently even when their AUC values are comparable, which often signals that the models are complementary and suggests that aggregating their output with an ensemble model may lead to improved accuracy.[20] Last, the AUC estimate derived by paired evaluation can be viewed as the average number of successes in a series of Bernoulli trials. While the trials are not independent and identically distributed (i.i.d.), the type I and type II errors are nevertheless well controlled (Figure S1), and the resulting AUC values will often follow a binomial distribution in practice, allowing for a reasonable approximation with a Gaussian distribution when the number of pairs is sufficiently large.

## The choice of test data has a profound effect on estimates of model performance

Breast cancer is a heterogeneous disease that is clinically subtyped based on the levels of expression of three receptors: tumors expressing estrogen and/or progesterone receptors are classified as HR+, tumors overexpressing and/or amplified for the HER2 receptor tyrosine kinase are classified as HER2 positive, and those lacking expression of these three genes are classified as triple-negative breast cancer (TNBC). In practice, clinical subtype determines how a cancer will be treated. Breast cancers are also classified based on gene expression profiles into four intrinsic molecular subtypes: luminal A, luminal B, basal, and HER2 enriched.[21,22] Molecular and clinical subtypes overlap but are non-identical. Given the high concordance between clinical and molecular subtypes in our cell line data (Figure S5), we followed the common practice of separating lines into luminal (HR+, HER2+) and basal (TNBC) molecular subtypes as a potential confounding variable.[23–26]

We considered a dataset recently collected in our laboratory that characterizes the sensitivity of 63 breast cancer cell lines of different subtypes to 72 small molecule drugs, with a focus on kinase inhibitors. The dataset comprises growth rate-corrected measures of drug sensitivity (GR values[27]), determined using a microscopy-based assay of cell proliferation and death,[28] and pre-treatment transcriptional and proteomic[29] profiles for each cell line. To demonstrate the effectiveness of paired evaluation, we considered a simple machine-learning setup, in which random forest regression models were trained to predict

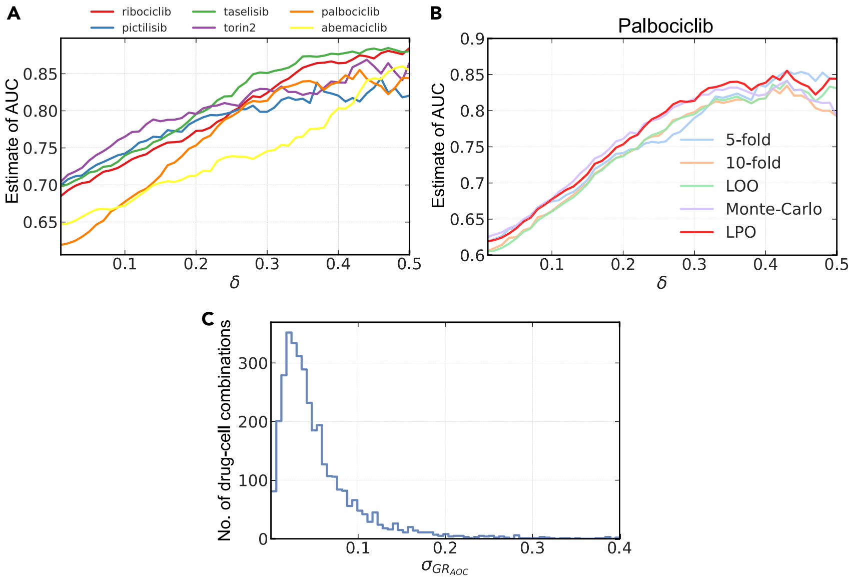


**Figure 2. The composition of the set of rankable pairs plays a crucial role in evaluating predictive models of drug response in breast cancer cell lines**

(A) The parameter $\delta$ defines the set of rankable cell line pairs, which are then used to estimate AUC of random forest models in LPOCV. Each model was trained to predict drug sensitivity from baseline mRNA expression. Shown are estimates of performance for six select compounds.

(B) Additional performance estimates for the drug palbociclib computed using 5-fold, 10-fold, and leave-one-out (LOO) cross-validation, as well as using Monte Carlo partitioning of the data into random 80%–20% train/test splits. The LPOCV estimates from (A) are included for reference.

(C) The distribution of standard deviation in $GR_{AOC}$ across technical triplicates for 3,400 drug-cell combinations. Predictive models are not expected to be able to distinguish two cell lines with $GR_{AOC}$ values that lie within the corresponding standard deviation since it represents measurement error.

See also Table S1.

drug sensitivity—measured as area over the growth rate curve ($GR_{AOC}$)—from the baseline mRNA expression of a set of preselected genes. Random forests were selected to model potential non-linear relationships in the data and because more complex models, e.g., neural networks, are overparameterized for the small number of data points (cell lines) in the dataset.

To account for possible measurement error, we considered a pair of cell lines to be rankable if the difference in the corresponding $GR_{AOC}$ labels was greater than a specified value of the meta-parameter $\delta$ (Figure 1B). We observed that the value of this meta-parameter had a dramatic impact on the estimate of model performance, with some estimates varying by as much as 20%. The trend was consistent across drugs (Figure 2A) and underlying cross-validation settings (Figure 2B). This finding reinforces the importance of choosing a test set that accurately represents potential future data that would be encountered by the predictor. Here, a large value of $\delta$ presents an "easy" prediction task, in which it is necessary only to distinguish cell line pairs having large differences in drug sensitivity. Such scenarios produce higher perceived model performance, but these estimates are artificially inflated relative to observed differences between cell lines in general and may not represent the true accuracy of the model.

To establish a reasonable value for $\delta$, we required that a corresponding model correctly ranks pairs of cell lines for which separation of $GR_{AOC}$ values (labels) was greater than experimental error. For a given drug-cell line combination, the experimental error was taken to be the standard deviation of $GR_{AOC}$ across three or four technical replicates. For any pair of cell lines, the larger of the two standard deviations was then used as the value for $\delta$ to determine if that pair was rankable. For most rankable pairs, this corresponded to a difference in $GR_{AOC}$ of $\delta < 0.3$ (the full range of $GR_{AOC}$ values in our data was $-0.7$ to 1.9), with the total number of rankable pairs on the order of hundreds for each drug (Figure 2C; Table S1). In the remainder of breast cancer data analyses, we applied paired evaluation in the context of LPOCV, where a separate model was trained for each test pair that was determined to be rankable using the above $\delta$ guidelines.

## Effect of breast cancer subtype on model performance

In our dataset, the dominant variance in gene expression data and drug sensitivity for multiple drugs was observed to align with molecular subtype (Figure S5), consistent with previous studies.[23,30] Thus, subtype represents a known complication in the analysis of breast cancer drug response, and we sought to evaluate its impact on estimates of model performance. To accomplish this, we broadly classified cells as either luminal or basal and compared AUC estimates computed with all rankable pairs against estimates derived using only those rankable pairs for which both cell lines were of the same subtype. For many drugs, we observed a decrease in estimated AUC when the evaluation was performed on subtype-matched pairs (Figure 3A), suggesting that the corresponding predictors had learned to recognize molecular subtype as a confounder. Next, we estimated the correlation between drug sensitivity and subtype using one-way ANOVA and observed that the resulting F-statistic was a good indicator of the difference between AUC estimates (Figure 3B). Our results confirm that learned models place more emphasis on the molecular subtype when it is indeed a good predictive feature of drug sensitivity. However, when prediction is limited to a single subtype, the models are frequently less accurate. The balance between accuracy across subtypes vs. within a subtype must therefore consider the way in which a model will be used. For example, if a drug is approved only for one subtype, then a subtype-specific model may be what is required.

To get a better understanding of the effect breast cancer subtype has on performance estimation, we considered six clinically relevant breast cancer drugs for closer examination (Table 1). Of these, alpelisib is currently approved for the treatment of HR+/HER2−metastatic breast cancers[31] and is in clinical trials for HER2+ patients. Palbociclib and abemaciclib are approved for use in the same metastatic HR+ breast cancers with current

CellPress
OPEN ACCESS

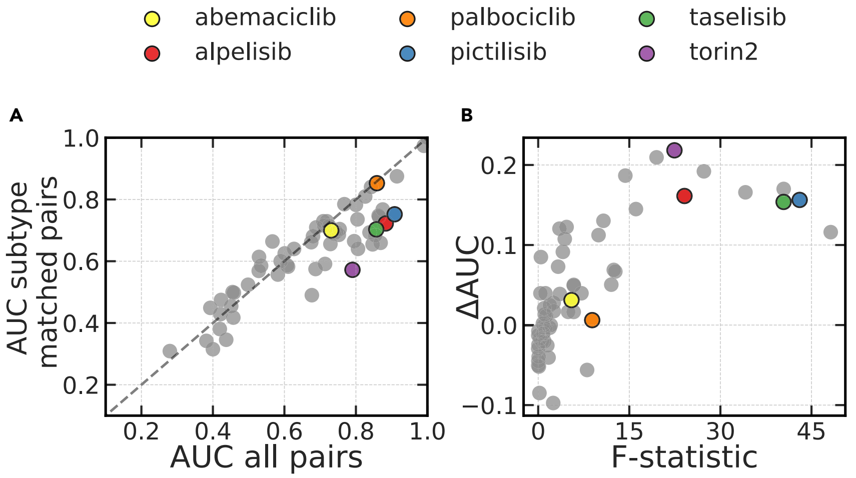


**Figure 3. Effect of breast cancer subtype on the estimates of prediction accuracy**

(A) AUC estimates calculated using subtype-matched (y axis) and all (x axis) rankable pairs. The dashed line represents all hypothetical scenarios where the two AUC estimates agree. Each dot corresponds to one of 72 drugs. A subset of drugs is highlighted for closer examination.

(B) The difference between AUC estimates in (B), computed as $\Delta AUC = AUC_{all} - AUC_{subtype}$ and plotted against matching one-way ANOVA to contrast $GR_{AOC}$ distributions across breast cancer subtypes as in (A). Each dot corresponds to one of 72 drugs.
See also Figure S7.

attempts to expand the indication to TNBC and HER2+ disease.[32] Consistent with these clinical indications, we found that basal and luminal cell lines responded differently to alpelisib, pictilisib, taselisib, and Torin2, while no significant difference in response was observed for palbociclib and abemaciclib (Figure S7).

In paired evaluation, the estimate of AUC was substantially lower for subtype-matched pairs when predicting sensitivity to alpelisib, pictilisib, taselisib, and Torin2 (Table 1 and Figure S6A), suggesting that the corresponding predictors had at least partially learned to recognize the molecular subtype. In contrast, no statistically significant difference was observed when comparing AUC estimates made using all pairs and subtype-matched pairs for palbociclib or abemaciclib (Table 1; Figure S6B), two drugs whose sensitivity was more weakly correlated with subtype (Figure S7).

An important aspect of paired evaluation is that it assesses the impact of known confounders on the prediction accuracy without modifying the original training data. This is in stark contrast to the traditional approaches of dealing with confounding variables, where the original measurements are perturbed to remove or reduce the impact of the confounders.[10,13,33] Taken together, our findings suggest that, when drug sensitivity is correlated with subtype, predictors implicitly learn features of the underlying subtypes. This may represent a desirable property in a setting where molecular subtype closely informs drug response.

## Detection and removal of outliers affects model interpretation

In the current setting, model interpretation primarily involves inspecting feature importance scores to pinpoint genes that play a crucial role in determining drug response and resistance. Since the presence of outliers in the training data can skew feature importance scores, we investigated the effects of outlier removal on model interpretation. We asked if any rankable pair was more likely to be ranked incorrectly by a model if it included specific data samples. We were specifically concerned about outliers that arose from measurement error or that were biologically very different from the norm. When predicting the sensitivity of breast cancer cell lines to Torin2, a polyselective mammalian target of rapamycin (mTOR) inhibitor,[34] we found that 526 out of 673 rankable pairs were ranked correctly by a random forest model (AUC estimate = 0.78). However, pairs containing the ZR7530 cell line were consistently ranked incorrectly (Figures 4A and 4B), suggesting that the cell line is an outlier. ZR7530 is a luminal cell line, and its gene expression profile clusters with profiles of other luminal cells (Figure S2). However, the cell line was more resistant to Torin2 than other luminal cell lines with a $GR_{AOC}$ value more similar to that of TNBC lines, explaining the observed misranking of pairs containing ZR7530.

Removing ZR7530 from the dataset reduced the total number of rankable pairs to 652, of which 524 were ranked correctly (Figure 4B), leading to a small improvement in estimated model accuracy (AUC estimate = 524/652 = 0.8). When we compared feature importance scores before and after the removal of ZR7530, we observed that *ERRB2* (*HER2*) increased in importance (Figures 4C and 4D), reconfirming that receptor status is heavily correlated with Torin2 response (Table 1). Similarly, we found that *MTOR*, which encodes a known target of Torin2,[35] also gains importance. More generally, these findings show that the feature importance of genes know to play an important role in breast cancer biology change when outliers are detected and removed in paired evaluation, and, at least in some cases, this increases interpretability.

We repeated the outlier analysis for all other drugs in our dataset and identified two other cases, corresponding to drugs E17 and palbociclib, for which sensitivity predictors consistently misranked pairs containing a specific cell line. In both cases, removing the outlier led to a higher estimate of AUC, but the effect on feature importance varied. In the case of E17, the removal of outlier cell line MGH312 led to a substantial drop in the importance *CDKN2C* (Figure S3). However, removing the outlier cell line HCC202 from a predictor of palbociclib sensitivity did not have any substantial impact on feature importance (Figure S4). These results demonstrate that the presence of outliers in a dataset can lead to a consistently incorrect ranking of pairs, and the removal of these outliers can increase, decrease, or have no effect on feature importance, making paired evaluation a useful tool for improving model interpretation.

## Disease severity in Alzheimer's decedents

AD is a chronic neurodegenerative disorder that leads to memory loss and dementia. The disease is characterized by

**Table 1. Effect of breast cancer subtype on model performance**

| Drug | | All rankable pairs | Subtype-matched pairs | p value |
|---|---|---|---|---|
| Alpelisib | ✓ | 337 | 80 | $7.67 \times 10^{-5}$ |
| | × | 30 | 24 | |
| | AUC | 0.92 | 0.77 | |
| Pictilisib | ✓ | 315 | 66 | $2.32 \times 10^{-4}$ |
| | × | 43 | 26 | |
| | AUC | 0.88 | 0.72 | |
| Taselisib | ✓ | 604 | 192 | $6.71 \times 10^{-9}$ |
| | × | 110 | 91 | |
| | AUC | 0.85 | 0.68 | |
| Torin2 | ✓ | 273 | 68 | $4.26 \times 10^{-8}$ |
| | × | 116 | 84 | |
| | AUC | 0.70 | 0.45 | |
| Palbociclib | ✓ | 367 | 176 | 0.5 |
| | × | 61 | 30 | |
| | AUC | 0.86 | 0.85 | |
| Abemaciclib | ✓ | 382 | 187 | 0.66 |
| | × | 177 | 82 | |
| | AUC | 0.68 | 0.70 | |

The p values were derived from one-sided Fisher's exact tests with the alternative hypothesis being that subtype-matched pairs were more likely to be misranked. See also Figure S6.

extracellular aggregates of the β-amyloid peptide and intracellular accumulation of hyperphosphorylated tau leading to neurofibrillary tangles (NFTs). Several recent studies—those from the Accelerating Medicines Partnership – Alzheimer's Disease (AMP-AD) program, for example[36]—have attempted to obtain molecular insight into disease mechanism using diverse -omic datasets obtained from patient specimens; these data include whole-genome sequencing, DNA methylation, mRNA and protein expression, and detailed clinical annotation.

Here, we consider the task of predicting disease severity from mRNA expression. We make use of the data collected by two joint longitudinal cohort studies, the Religious Orders Study (ROS) and the Memory and Aging Project (MAP), that comprise over 200 bulk RNA-seq profiles of postmortem brain specimens, along with matching pathology annotations.[37,38] We group data points into three categories based on Braak staging[39,40]: mild (Braak 1–2), moderate (Braak 3–4), and severe (Braak 5–6).

AD progression takes place on a timescale of years, and disease severity is strongly correlated with a patient's age of death (AOD) (Figure 5A). An important question is whether a predictor trained to recognize disease severity has instead learned to predict age, a situation that can lead to an overinflated estimate of performance and affect the interpretation of the genes and weights that make up the model. To address this, we used paired evaluation as a non-parametric way of evaluating the effect of a known confounder on performance estimates; this involved contrasting confounder-matched and confounder-mismatched rankable pairs. As with breast cancer data above, we used paired evaluation in an LPOCV setting because of the natural integration between the two.

In our breast cancer analysis, the labels were continuous (varying from 0 to 1 $GR_{AOC}$), and the confounder was represented by a discrete variable (basal or luminal subtype). The opposite is true of ROSMAP data; the labels are discrete, resulting in a straightforward definition of rankable pairs: two brain specimens are rankable if they have distinct Braak stage ranges. Conversely, age is a continuous variable that is censored at 90 years old (y.o.). The censoring provides a natural inflection point for determining whether two data points are matched in age, giving rise to two evaluation scenarios (Figure 5B). In a scenario focused on mild disease (a mild-centric scenario), each individual who passed away before the age of 90 years with mild disease was paired with individuals who had severe disease and was either closest in age (AOD matched) or was chosen at random from the censored category of 90+ y.o. (AOD confounded). Similarly, a severe-centric scenario pairs each 90+ y.o. patient who passed away with severe AD with a patient who had mild disease and was either randomly selected from the same 90+ y.o. category (AOD matched) or the youngest patient in the cohort (AOD mismatched). As a reference point, we also considered all possible rankable pairs.

In both mild-centric and severe-centric scenarios, each data point was associated with two rankable pairs that represent the minimal and maximal separation along the confounding variable (Figure 5B). To demonstrate that paired evaluation is agnostic to the underlying machine-learning algorithm, we switched from random forests to logistic regression and trained models to recognize disease severity from the corresponding mRNA expression profiles, applying paired evaluation to estimate model performance with each set of rankable pairs. We found that the models performed similarly for AOD-matched and AOD-confounded pairs (Figure 5C) and that both performance estimates were consistent with the one derived on all rankable pairs (mild-centric AUC = 0.87, severe-centric AUC = 0.85). Similar performance trends were also observed for other classification tasks (Figure S8), with a single exception being a mild-centric comparison of mild and moderate disease severity, where predictors may have learned to recognize the AOD. This trend is expected, given the relative difficulty of distinguishing among the early stages of the disease in a younger population.[41]

The analysis reveals that the presence of confounders does not necessitate that a predictor will learn to recognize them instead of the variable of interest. Paired evaluation provides a simple way to detect whether such situations occur and can facilitate decisions about when it is necessary to correct for confounding variables.

## DISCUSSION

In this work, we extend paired evaluation, a method for deriving detailed landscapes of predictor performance for machine-learned models based on the concept of rankable pairs of datapoints. We show how systematic pairing of data points can account for known confounders and identify outliers. We also show that statistical significance of model comparison can be maintained using standard tests based on contingency tables. While we made use of LPOCV in the current work due to its natural integration with paired evaluation, the proposed methodology can be applied in any cross-validation setting that allows

CellPress
OPEN ACCESS

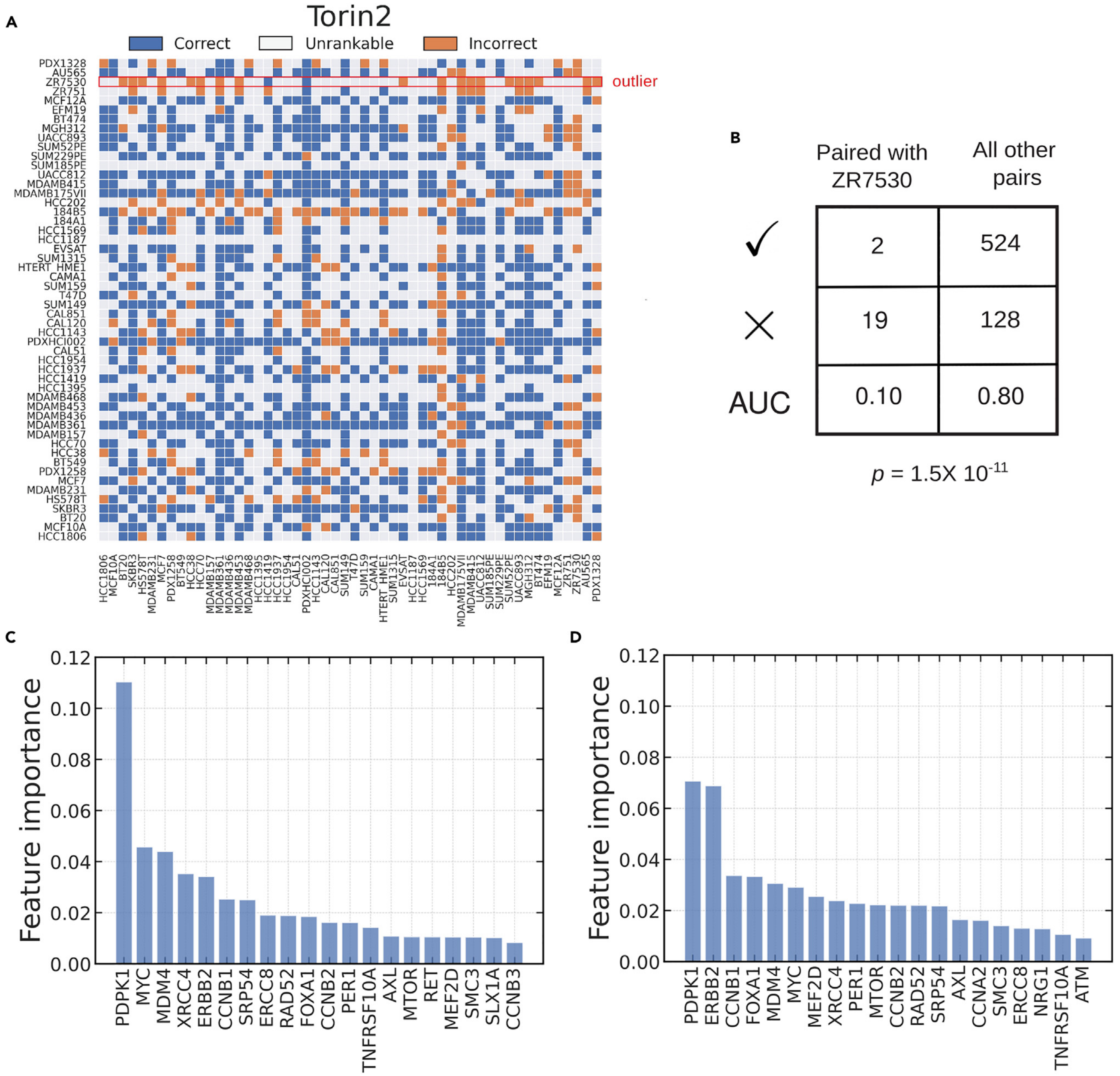


**Figure 4. Paired evaluation detects outlier cell lines in the context of sensitivity to Torin2**

(A) A performance landscape over all possible pairs of cell lines. A pair is colored blue if it was correctly ranked by the predictor and orange otherwise. Pairs that were not considered rankable because the corresponding $GR_{AOC}$ values were not separated by the $\delta$ threshold are shown in gray.

(B) A 2 × 2 contingency table tallying correctly and incorrectly ranked pairs with and without the cell line ZR7530. The corresponding p value was computed using a one-sided Fisher's exact test with the alternative hypothesis being that pairs including ZR7530 were more likely to be misranked.

(C) Feature importance scores associated with a predictor trained on all cell lines. Shown are the top 20 features.

(D) Feature importance scores computed after removing the outlier ZR7530 and retraining the predictor on the remaining cell lines.

See also Figure S2.

for a comparison of predictions across test folds. An efficient $O(n \log n)$ implementation of paired evaluation using inversion counting (see experimental procedures) allows the method to scale easily to datasets with millions of samples (Figure S9).

The choice of test data can have a dramatic effect on estimates of model performance.[1] To get an accurate performance estimate, a test set must be a faithful representation of future data that a predictor might encounter in deployment. We therefore recommend that rankable pairs be defined using experimental knowledge and domain expertise. For example, in regression problems, the choice of a minimal difference (in a continuous variable) for a pair to be rankable ($\delta$) could be based

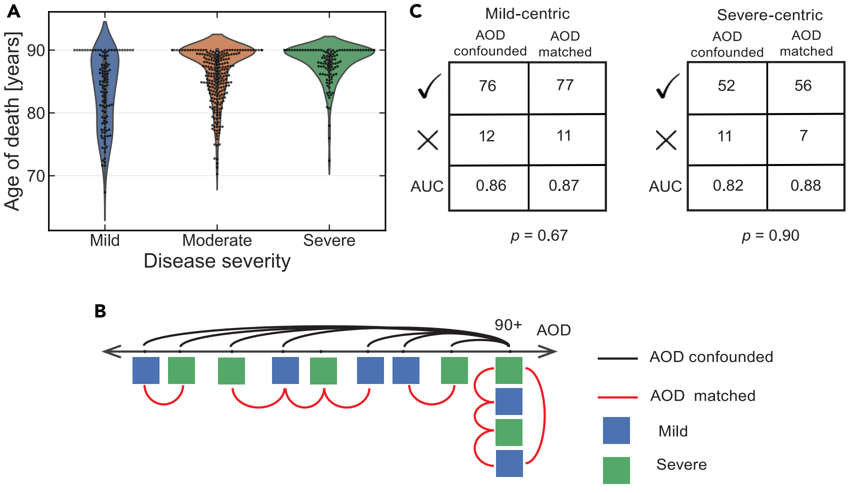


**Figure 5. Paired evaluation reveals that models trained to recognize disease stage are not confounded by age**

(A) The distribution of age of death (AOD) for patients who were diagnosed with mild, moderate, or severe AD during postmortem pathology analysis.

(B) Schematic representation of rankable pairs, selected to be either confounder matched (red) or mismatched (black). Each patient is represented by a square, colored according to the corresponding pathology annotation. The value of AOD is censored at 90 years of age in the dataset.

(C) 2 × 2 contingency tables showing the correctly and incorrectly ranked test pairs for AOD-confounded and AOD-matched scenarios. The p value was computed using a one-sided Fisher's exact test with the alternative hypothesis being that AOD-matched pairs were more likely to be misranked by the model.

See also Figure S8.

on variance across biological or technical replicates (Figure 1B); two data points that fall within this variance are deemed indistinguishable. Confounding and lurking variables are ubiquitous, but their presence may not be a drawback if they are biologically relevant and can assist in model interpretation. In the case of breast cancer cell lines, the difference between $HR^+$, $HER2^+$, and TNBC status confounds modeling of drug sensitivity but is informative for drugs that inhibit HR and HER2.[30] Conversely, a predictor that unintentionally learns to recognize what institution a subset of data was collected at in a multi-center study[9] is unlikely to produce meaningful biological insight. Because confounders can have either positive or adverse effects on model interpretation, it is imperative to know when predictors have learned to recognize confounders. We showed that paired evaluation is an effective, non-parametric method to detect this through simple comparison of performance values computed on confounder-matched vs. confounder-mismatched pairs. Importantly, paired evaluation achieves this without modifying the original data and without the need to train additional models on subsets of data stratified by the confounders of interest.

Our study has several limitations. In its present formulation, defining confounder-matched rankable pairs requires that the confounder values are known; however, many datasets may have unknown or unmeasured lurking variables that introduce unwanted batch effects.[7] To evaluate the effect of these hidden variables on the estimate of performance using paired evaluation, a user would first have to detect them using an external method. We also expect that information about hidden batch effects may be encoded in pairwise comparison of data points. Our future work will extend the outlier detection to identify groups of samples that exhibit similar misranking patterns as a method for approximating shared unobserved characteristics. While paired evaluation can detect situations in which confounders affected model training, the method provides no intrinsic means to correct for this effect, since the original training data are not modified. Furthermore, it is not trivial to delineate what aspect of model interpretation (e.g., feature importance) aligns with a confounder versus the variable of interest, even when paired evaluation signals that a predictor learned to recognize that confounder. Thus, paired evaluation represents the initial step in identifying potentially problematic confounder variables and outlier samples, but resolution of these may require other methods.

## EXPERIMENTAL PROCEDURES

### Resource availability

#### *Lead contact*

Further information and requests for resources and reagents should be directed to and will be fulfilled by the lead contact, Artem Sokolov (artem_sokolov@hms.harvard.edu).

#### *Materials availability*

This study did not generate new unique reagents.

#### *Data and code availability*

The results published here are in part based on data obtained from the AD Knowledge Portal.[42] We used the data collected at our laboratory to train and evaluate predictors of drug sensitivity in breast cancer cell lines.[43] An efficient $O(n \log n)$ implementation of paired evaluation, as well as a Python module for executing paired evaluation in an LPOCV setting, is publicly available as a GitHub repository.[44]

### Estimation of AUC

We consider a pair of samples $i$ and $j$ to be rankable if their labels ($y_i$ and $y_j$, respectively) satisfy

$$f(y_i, y_j) \geq \delta_{i,j} \qquad \text{(Equation 1)}$$

where $f$ is a distance function and $\delta_{i,j}$ is the minimum necessary threshold of label separation. In classification, $f$ was set to be an indicator function returning 0 if the arguments are identical and 1 otherwise, while $\delta_{i,j}$ was set to 0.5 for all $(i,j)$. In linear regression, $f$ was the L1-norm distance $|y_i - y_j|$ in the label space, and $\delta_{i,j} = \max(\sigma_i, \sigma_j)$ was taken to be the expected measurement error, estimated by the standard deviations $\sigma_i$ and $\sigma_j$ computed across technical replicates.

Given the space of rankable pairs $R$, AUC is estimated by

$$AUC = \sum_{i,j \in R} \frac{p_{ij}}{|R|} \qquad \text{(Equation 2)}$$

where $p_{ij}$ is an indicator variable that takes on the value of 1 when the pair of samples $i,j$ is correctly ranked by the predictor, and 0 otherwise.

### Applying paired evaluation in cross-validation

To determine if a rankable pair is ranked correctly by a model, paired evaluation requires that the corresponding predictor assigns scores to both samples in the pair. The scores can be probabilities that the input sample belongs to the

positive class in binary classification, ranks in a recommender system, or predictions of real-valued measurements. Since pairwise comparison of scores by paired evaluation is a separate task, the scores from multiple test partitions can be pooled together. This allows paired evaluation to be applied in any cross-validation setting, including leave-one-out cross-validation, as long as pooled prediction scores can be ordered by their corresponding representation (e.g., predicted hazard ratio by a patient survival model). If cross-validation produces multiple scores for a single sample, e.g., through repeated random partitioning into 80% train/20% test splits, we use the score average for that sample in paired evaluation. An exception to the above rule is LPOCV, where each rankable pair can be evaluated directly, without the need for score averaging.

## Efficient implementation of paired evaluation using inversion counting

Given a test dataset of n samples with the corresponding labels $y_1 \leq y_2 \leq \ldots \leq y_n$ that designate the desired ranking of samples, paired evaluation counts the number of pairs that are actually ranked correctly when the samples are sorted based on the scores produced by a given machine-learning model. This is a well-known problem in computer science called inversion counting and an $O(n \log n)$ implementation is given by the following modified merge sort algorithm:

1. Sort the samples based on the scores produced by a model
2. Let $z_i$ designate the label of the sample in the $i^{th}$ position
3. We define a function *count(l, r)*, where *l* and *r* are the left and right endpoints of an interval along the sorted list. Inside this function,
   a. Initialize *inv = 0*
   b. Terminate recursion if $r < l$
   c. Compute the midpoint of the interval: $m = floor((l + r) / 2)$
   d. Recurse on each half: inv = inv + count(l, m) + count(m + 1, r)
   e. Count the inversions using the standard merge sort loop, by initializing pointers i = l, j = m + 1 and traversing the two halves of the interval, while $i \leq m$ and $j \leq r$ is satisfied
      i. During the traversal, no inversions are counted whenever $z_i \leq z_j$
      ii. Otherwise, everything in the left half of the interval between the current pointer *i* and the midpoint is an inversion relative to *j*: inv = inv + m − i +1
   f. Return the overall tally *inv* from the function
4. The total number of correctly ranked pairs is the number of rankable pairs minus the total number of inversions. Thus, AUC is given by $(|R| - count(1, n))/|R|$, where *R* is the set of all rankable pairs as before.

## Outlier detection

We define the sample-specific AUC for the *k*-th sample as,

$$AUC_k = \sum_{i,j \in R_k} \frac{p_{ij}}{|R_k|} \quad \text{(Equation 3)}$$

where $R_k \subset R$ is the subset of all rankable pairs that include the sample *k*, and $p_{i,j}$ has the same interpretation as in Equation 2. Samples with significantly lower $AUC_k$ than the overall AUC were considered to be potential outliers and inspected in more detail to decide whether they warrant an exclusion from the study. As with method comparison (Figure 1C), statistical significance was assessed by constructing a two-by-two contingency table cataloging whether a given pair of samples is in $R_k$ and whether that pair was ranked correctly by the corresponding model. Fisher's exact test was used to determine whether pairs in $R_k$ were ranked correctly significantly more often than pairs not in $R_k$.

## Robust evaluation of predictors in the presence of confounders

To measure the effect of known confounders on the estimate of model performance, we considered a subset of rankable pairs where the difference in the confounder values was minimal. For discrete confounding variables (e.g., breast cancer subtype), the values were matched exactly. For continuous variables, we selected a single rankable pair per sample, such that the difference between the two values of the confounder was minimized. A possible unexplored alternative was to consider all samples that fell within a certain predefined "match" window for a given index sample. In all cases, we refer to resulting subsets of rankable pairs as confounder matched and the remaining rankable pairs as confounder mismatched.

If AUC estimated on confounder-matched pairs was significantly lower than its equivalent derived from confounder-mismatched pairs, then this was interpreted as a strong indication that the corresponding predictor has learned to recognize the confounder instead of the variable of interest. Statistical significance was again assessed with a Fisher's exact test applied to a two-by-two contingency table cataloging whether rankable pairs were more likely to be ranked correctly if they are confounder matched or confounder mismatched.

## SUPPLEMENTAL INFORMATION

Supplemental information can be found online at https://doi.org/10.1016/j.patter.2023.100791.

## ACKNOWLEDGMENTS

We acknowledge support by the NIH Illuminating the Druggable Genome program (U24-DK116204), the NCI grant U54-CA225088, and the NIA grant R01 AG058063. ROSMAP study data were provided by the Rush Alzheimer's Disease Center, Rush University Medical Center, Chicago. We thank Kartik Subramanian and Juliann Tefft for helpful discussions.

## AUTHOR CONTRIBUTIONS

Conceptualization, A.S.; methodology, M.K.N. and A.S.; investigation, M.K.N., C.E.M., and A.S.; writing – original draft, M.K.N., C.E.M., P.K.S., and A.S.; writing – review & editing, M.K.N. and A.S.; funding acquisition, A.S. and P.K.S.; resources, A.S. and P.K.S.; supervision, A.S. and P.K.S.

## DECLARATION OF INTERESTS

P.K.S. is a member of the SAB or BOD member of Applied Biomath, RareCyte Inc., and Glencoe Software. P.K.S. is also a member of the NanoString SAB. In the last 5 years, the Sorger laboratory has received research funding from Novartis and Merck. A.S. is an employee at FL84, Inc., a subsidiary of Flagship Pioneering.

## INCLUSION AND DIVERSITY

We support inclusive, diverse, and equitable conduct of research.

Received: February 6, 2023
Revised: March 27, 2023
Accepted: June 8, 2023
Published: July 7, 2023

## REFERENCES

1. D'Amour, A., Heller, K., Moldovan, D., Adlam, B., Alipanahi, B., Beutel, A., Chen, C., Deaton, J., Eisenstein, J., Hoffman, M.D., et al. (2022). Underspecification presents challenges for credibility in modern machine learning. J. Mach. Learn. Res. *23*, 10237–10297.

2. Geman, S., Bienenstock, E., and Doursat, R. (1992). Neural networks and the bias/variance dilemma. Neural Comput. *4*, 1–58. https://doi.org/10.1162/neco.1992.4.1.1.

3. Stone, M. (1974). Cross-validatory choice and assessment of statistical predictions. J. Roy. Stat. Soc. B *36*, 111–133. https://doi.org/10.1111/j.2517-6161.1974.tb00994.x.

4. Geisser, S. (1975). The predictive sample reuse method with applications. J. Am. Stat. Assoc. *70*, 320–328. https://doi.org/10.2307/2285815.

5. Efron, B., and Tibshirani, R. (1997). Improvements on cross-validation: the .632+ bootstrap method. J. Am. Stat. Assoc. *92*, 548–560. https://doi.org/10.2307/2965703.

6. Dai, X., Cheng, H., Bai, Z., and Li, J. (2017). Breast cancer cell line classification and its relevance with breast tumor subtyping. J. Cancer *8*, 3131–3141. https://doi.org/10.7150/jca.18457.

7. Leek, J.T., and Storey, J.D. (2007). Capturing heterogeneity in gene expression studies by surrogate variable analysis. PLoS Genet. *3*, 1724–1735. https://doi.org/10.1371/journal.pgen.0030161.

8. Parker, B.J., Günter, S., and Bedo, J. (2007). Stratification bias in low signal microarray studies. BMC Bioinf. *8*, 326. https://doi.org/10.1186/1471-2105-8-326.

9. Niepel, M., Hafner, M., Mills, C.E., Subramanian, K., Williams, E.H., Chung, M., Gaudio, B., Barrette, A.M., Stern, A.D., Hu, B., et al. (2019). A multi-center study on the reproducibility of drug-response assays in mammalian cell lines. Cell Syst. *9*, 35–48.e5. https://doi.org/10.1016/j.cels.2019.06.005.

10. Johnson, W.E., Li, C., and Rabinovic, A. (2007). Adjusting batch effects in microarray expression data using empirical Bayes methods. Biostatistics *8*, 118–127. https://doi.org/10.1093/biostatistics/kxj037.

11. Risso, D., Ngai, J., Speed, T.P., and Dudoit, S. (2014). Normalization of RNA-seq data using factor analysis of control genes or samples. Nat. Biotechnol. *32*, 896–902. https://doi.org/10.1038/nbt.2931.

12. Smyth, G.K. (2004). Linear models and empirical bayes methods for assessing differential expression in microarray experiments. Stat. Appl. Genet. Mol. Biol. *3*, Article3. https://doi.org/10.2202/1544-6115.1027.

13. Nygaard, V., Rødland, E.A., and Hovig, E. (2016). Methods that remove batch effects while retaining group differences may lead to exaggerated confidence in downstream analyses. Biostatistics *17*, 29–39. https://doi.org/10.1093/biostatistics/kxv027.

14. Rosset, S., Perlich, C., and Zadrozny, B. (2005). Ranking-based evaluation of regression models. In Fifth IEEE International Conference on Data Mining (ICDM'05), p. 8. https://doi.org/10.1109/ICDM.2005.126.

15. Bradley, A.P. (1997). The use of the area under the ROC curve in the evaluation of machine learning algorithms. Pattern Recogn. *30*, 1145–1159. https://doi.org/10.1016/S0031-3203(96)00142-2.

16. Airola, A., Pahikkala, T., Waegeman, W., De Baets, B., and Salakoski, T. (2011). An experimental comparison of cross-validation techniques for estimating the area under the ROC curve. Comput. Stat. Data Anal. *55*, 1828–1844. https://doi.org/10.1016/j.csda.2010.11.018.

17. Smith, G.C.S., Seaman, S.R., Wood, A.M., Royston, P., and White, I.R. (2014). Correcting for optimistic prediction in small data sets. Am. J. Epidemiol. *180*, 318–324. https://doi.org/10.1093/aje/kwu140.

18. Montoya Perez, I., Airola, A., Boström, P.J., Jambor, I., and Pahikkala, T. (2019). Tournament leave-pair-out cross-validation for receiver operating characteristic analysis. Stat. Methods Med. Res. *28*, 2975–2991. https://doi.org/10.1177/0962280218795190.

19. Dietterich, T.G. (1998). Approximate statistical tests for comparing supervised classification learning algorithms. Neural Comput. *10*, 1895–1923. https://doi.org/10.1162/089976698300017197.

20. van der Laan, M.J., Polley, E.C., and Hubbard, A.E. (2007). Super learner. Stat. Appl. Genet. Mol. Biol. *6*, Article25. https://doi.org/10.2202/1544-6115.1309.

21. Perou, C.M., Sørlie, T., Eisen, M.B., van de Rijn, M., Jeffrey, S.S., Rees, C.A., Pollack, J.R., Ross, D.T., Johnsen, H., Akslen, L.A., et al. (2000). Molecular portraits of human breast tumours. Nature *406*, 747–752. https://doi.org/10.1038/35021093.

22. Sørlie, T., Perou, C.M., Tibshirani, R., Aas, T., Geisler, S., Johnsen, H., Hastie, T., Eisen, M.B., van de Rijn, M., Jeffrey, S.S., et al. (2001). Gene expression patterns of breast carcinomas distinguish tumor subclasses with clinical implications. Proc. Natl. Acad. Sci. USA *98*, 10869–10874. https://doi.org/10.1073/pnas.191367098.

23. Neve, R.M., Chin, K., Fridlyand, J., Yeh, J., Baehner, F.L., Fevr, T., Clark, L., Bayani, N., Coppe, J.-P., Tong, F., et al. (2006). A collection of breast cancer cell lines for the study of functionally distinct cancer subtypes. Cancer Cell *10*, 515–527. https://doi.org/10.1016/j.ccr.2006.10.008.

24. Tang, P., Wang, J., and Bourne, P. (2008). Molecular classifications of breast carcinoma with similar terminology and different definitions: are they the same? Hum. Pathol. *39*, 506–513. https://doi.org/10.1016/j.humpath.2007.09.005.

25. Lehmann, B.D., Bauer, J.A., Chen, X., Sanders, M.E., Chakravarthy, A.B., Shyr, Y., and Pietenpol, J.A. (2011). Identification of human triple-negative breast cancer subtypes and preclinical models for selection of targeted therapies. J. Clin. Invest. *121*, 2750–2767. https://doi.org/10.1172/JCI45014.

26. Dai, X., Li, T., Bai, Z., Yang, Y., Liu, X., Zhan, J., and Shi, B. (2015). Breast cancer intrinsic subtype classification, clinical use and future trends. Am. J. Cancer Res. *5*, 2929–2943.

27. Hafner, M., Niepel, M., Chung, M., and Sorger, P.K. (2016). Growth rate inhibition metrics correct for confounders in measuring sensitivity to cancer drugs. Nat. Methods *13*, 521–527. https://doi.org/10.1038/nmeth.3853.

28. Mills, C.E., Subramanian, K., Hafner, M., Niepel, M., Gerosa, L., Chung, M., Victor, C., Gaudio, B., Yapp, C., Nirmal, A.J., et al. (2022). Multiplexed and reproducible high content screening of live and fixed cells using the Dye Drop method. Preprint at bioRxiv. https://doi.org/10.1101/2021.08.27.457854.

29. Kalocsay, M., Berberich, M.J., Everley, R.A., Nariya, M.K., Chung, M., Gaudio, B., Victor, C., Bradshaw, G.A., Hafner, M., Sorger, P.K., et al. (2020). Data Descriptor: proteomic profiling across breast cancer cell lines and models. Preprint at bioRxiv. https://doi.org/10.1101/2020.12.15.422823.

30. Heiser, L.M., Sadanandam, A., Kuo, W.-L., Benz, S.C., Goldstein, T.C., Ng, S., Gibb, W.J., Wang, N.J., Ziyad, S., Tong, F., et al. (2012). Subtype and pathway specific responses to anticancer compounds in breast cancer. Proc. Natl. Acad. Sci. USA *109*, 2724–2729. https://doi.org/10.1073/pnas.1018854108.

31. André, F., Ciruelos, E., Rubovszky, G., Campone, M., Loibl, S., Rugo, H.S., Iwata, H., Conte, P., Mayer, I.A., Kaufman, B., et al. (2019). Alpelisib for PIK3CA-mutated, hormone receptor–positive advanced breast cancer. N. Engl. J. Med. *380*, 1929–1940. https://doi.org/10.1056/NEJMoa1813904.

32. Pernas, S., Tolaney, S.M., Winer, E.P., and Goel, S. (2018). CDK4/6 inhibition in breast cancer: current practice and future directions. Ther. Adv. Med. Oncol. *10*, 1758835918786451. https://doi.org/10.1177/1758835918786451.

33. Molania, R., Foroutan, M., Gagnon-Bartsch, J.A., Gandolfo, L.C., Jain, A., Sinha, A., Olshansky, G., Dobrovic, A., Papenfuss, A.T., and Speed, T.P. (2023). Removing unwanted variation from large-scale RNA sequencing data with PRPS. Nat. Biotechnol. *41*, 82–95. https://doi.org/10.1038/s41587-022-01440-w.

34. Chopra, S.S., Jenney, A., Palmer, A., Niepel, M., Chung, M., Mills, C., Sivakumaren, S.C., Liu, Q., Chen, J.-Y., Yapp, C., et al. (2020). Torin2 exploits replication and checkpoint vulnerabilities to cause death of PI3K-activated triple-negative breast cancer cells. Cells *10*, 66–81.e11. https://doi.org/10.1016/j.cels.2019.11.001.

35. Liu, Q., Xu, C., Kirubakaran, S., Zhang, X., Hur, W., Liu, Y., Kwiatkowski, N.P., Wang, J., Westover, K.D., Gao, P., et al. (2013). Characterization of Torin2, an ATP-competitive inhibitor of mTOR, ATM, and ATR. Cancer Res. *73*, 2574–2586. https://doi.org/10.1158/0008-5472.CAN-12-1702.

36. Hodes, R.J., and Buckholtz, N. (2016). Accelerating medicines partnership: Alzheimer's disease (AMP-AD) knowledge portal aids Alzheimer's drug discovery through open data sharing. Expert Opin. Ther. Targets *20*, 389–391. https://doi.org/10.1517/14728222.2016.1135132.

37. Bennett, D.A., Schneider, J.A., Arvanitakis, Z., and Wilson, R.S. (2012). Overview and findings from the religious orders study. Curr. Alzheimer Res. *9*, 628–645. https://doi.org/10.2174/156720512801322573.

38. De Jager, P.L., Ma, Y., McCabe, C., Xu, J., Vardarajan, B.N., Felsky, D., Klein, H.-U., White, C.C., Peters, M.A., Lodgson, B., et al. (2018). A multi-omic atlas of the human frontal cortex for aging and Alzheimer's disease research. Sci. Data *5*, 180142. https://doi.org/10.1038/sdata.2018.142.

39. Jouanne, M., Rault, S., and Voisin-Chiret, A.-S. (2017). Tau protein aggregation in Alzheimer's disease: an attractive target for the development of novel therapeutic agents. Eur. J. Med. Chem. *139*, 153–167. https://doi.org/10.1016/j.ejmech.2017.07.070.

CellPress
OPEN ACCESS

40. Braak, H., and Braak, E. (1991). Neuropathological stageing of Alzheimer-related changes. Acta Neuropathol. *82*, 239–259. https://doi.org/10.1007/BF00308809.

41. Rodriguez, S., Hug, C., Todorov, P., Moret, N., Boswell, S.A., Evans, K., Zhou, G., Johnson, N.T., Hyman, B.T., Sorger, P.K., et al. (2021). Machine learning identifies candidates for drug repurposing in Alzheimer's disease. Nat. Commun. *12*, 1033. https://doi.org/10.1038/s41467-021-21330-0.

42. Greenwood, A.K., Montgomery, K.S., Kauer, N., Woo, K.H., Leanza, Z.J., Poehlman, W.L., Gockley, J., Sieberts, S.K., Bradic, L., Logsdon, B.A., et al. (2020). The AD knowledge portal: a repository for multi-omic data on Alzheimer's disease and aging. Curr. Protoc. Hum. Genet. *108*, e105. https://doi.org/10.1002/cphg.105.

43. Sokolov, A., and Nariya, M. (2023). labsyspharm/brca-profiling: evaluating the capacity of gene sets to predict drug response in breast cancer cell lines. Zenodo. https://doi.org/10.5281/zenodo.8001724.

44. Sokolov, A., and Nariya, M. (2023). Labsyspharm/Paired-Eval: Paired Evaluation of Machine Learning Models. Zenodo. https://doi.org/10.5281/zenodo.8001730.

**Patterns, Volume *4***

## Supplemental information

# Paired evaluation of machine-learning models characterizes effects of confounders and outliers

**Maulik K. Nariya, Caitlin E. Mills, Peter K. Sorger, and Artem Sokolov**

# Supplementary information

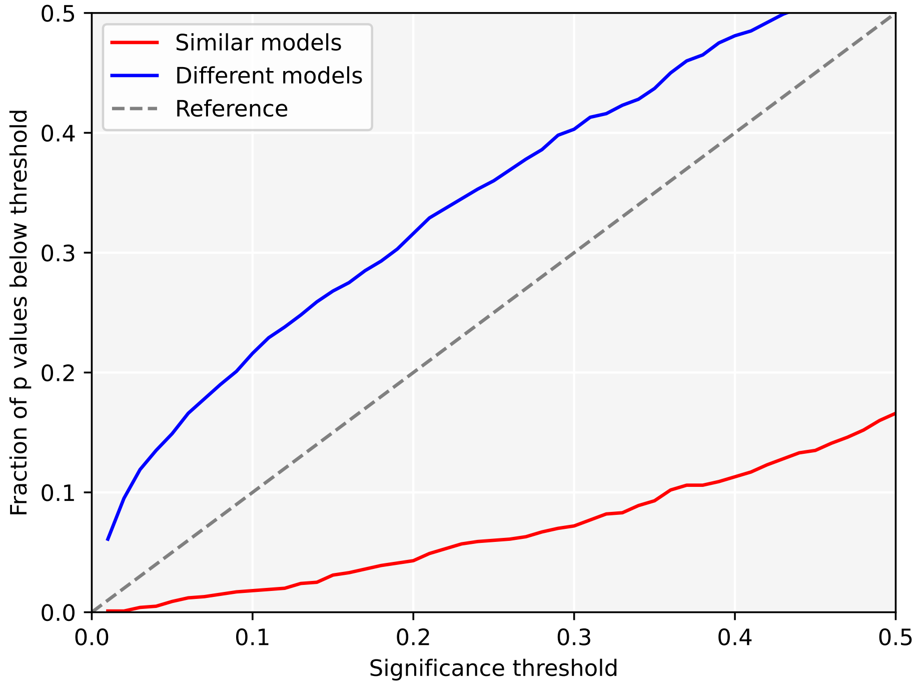


**Figure S1. The probability of paired evaluation rejecting the null hypothesis that two models have the same performance**. Shown is the fraction of p values from 1,000 two-sided Fisher's Exact tests that fall below a given significance threshold. Each p value was derived on a random 80/20 train/test split of the breast cancer dataset (see Results) by comparing the performance of two random forest models trained with identical hyperparameter values (red) or one random forest model and one linear regression model (blue). The expected "no information" rate is presented as a dashed line for reference.

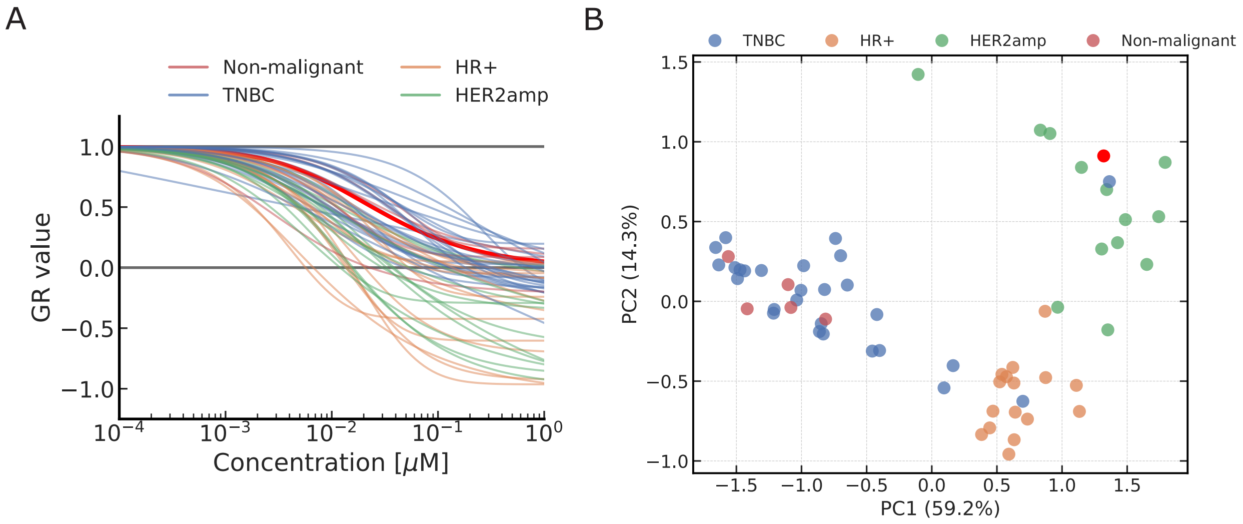


**Figure S2. The location of ZR7530 data relative to other cell lines. A.)** Growth rates curves for torin2 across all breast cancer cell lines. **B.)** Principal components analysis of the baseline RNA-seq data, computed in the space of the top 20 most important genes (Figure 3). The "outlier" cell line ZR7530 is highlighted in red, all other cell lines are colored by their subtypes.

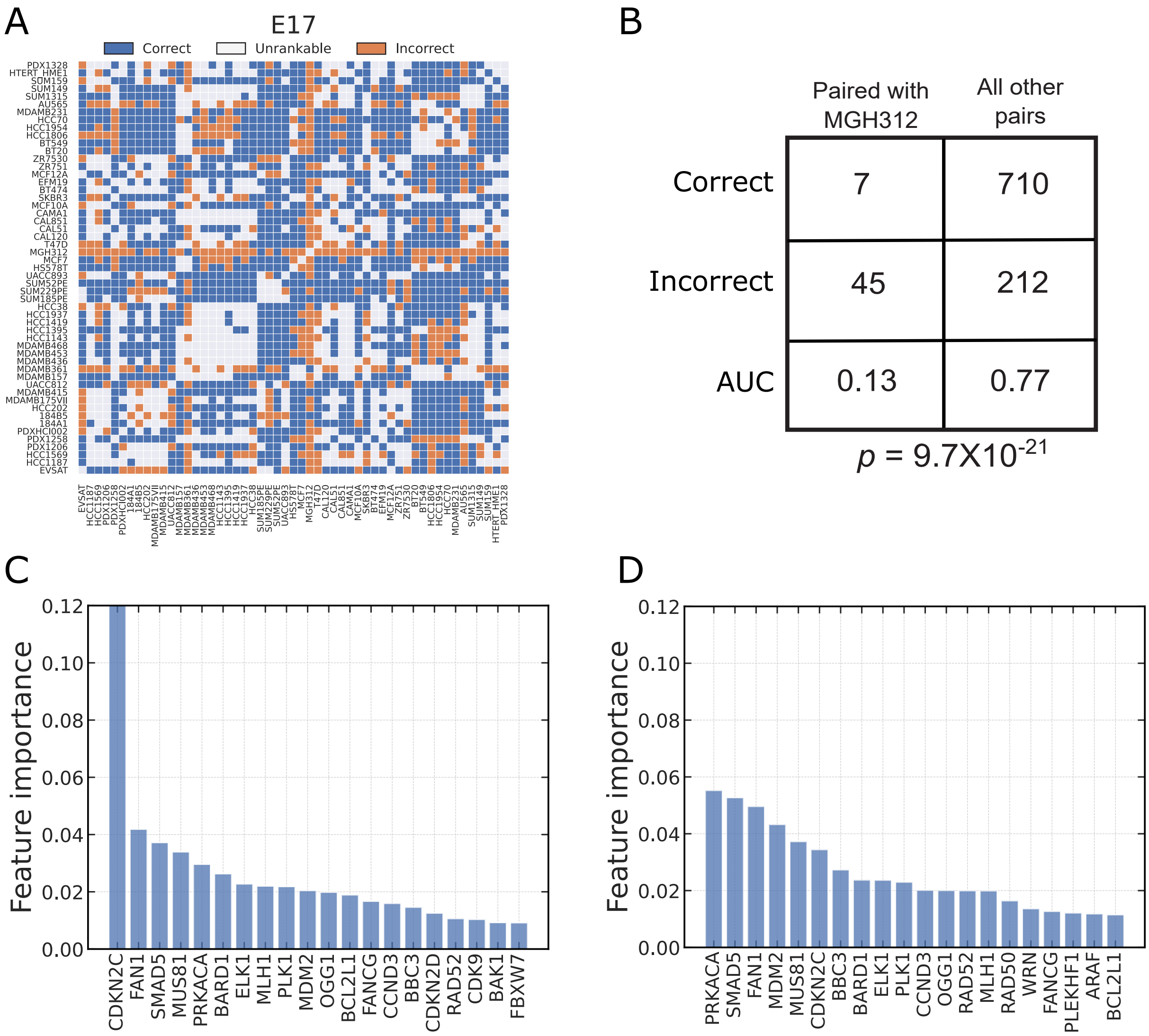


**Figure S3. Outlier detection for E17.** The interpretation of all panels is analogous to Figure 3.

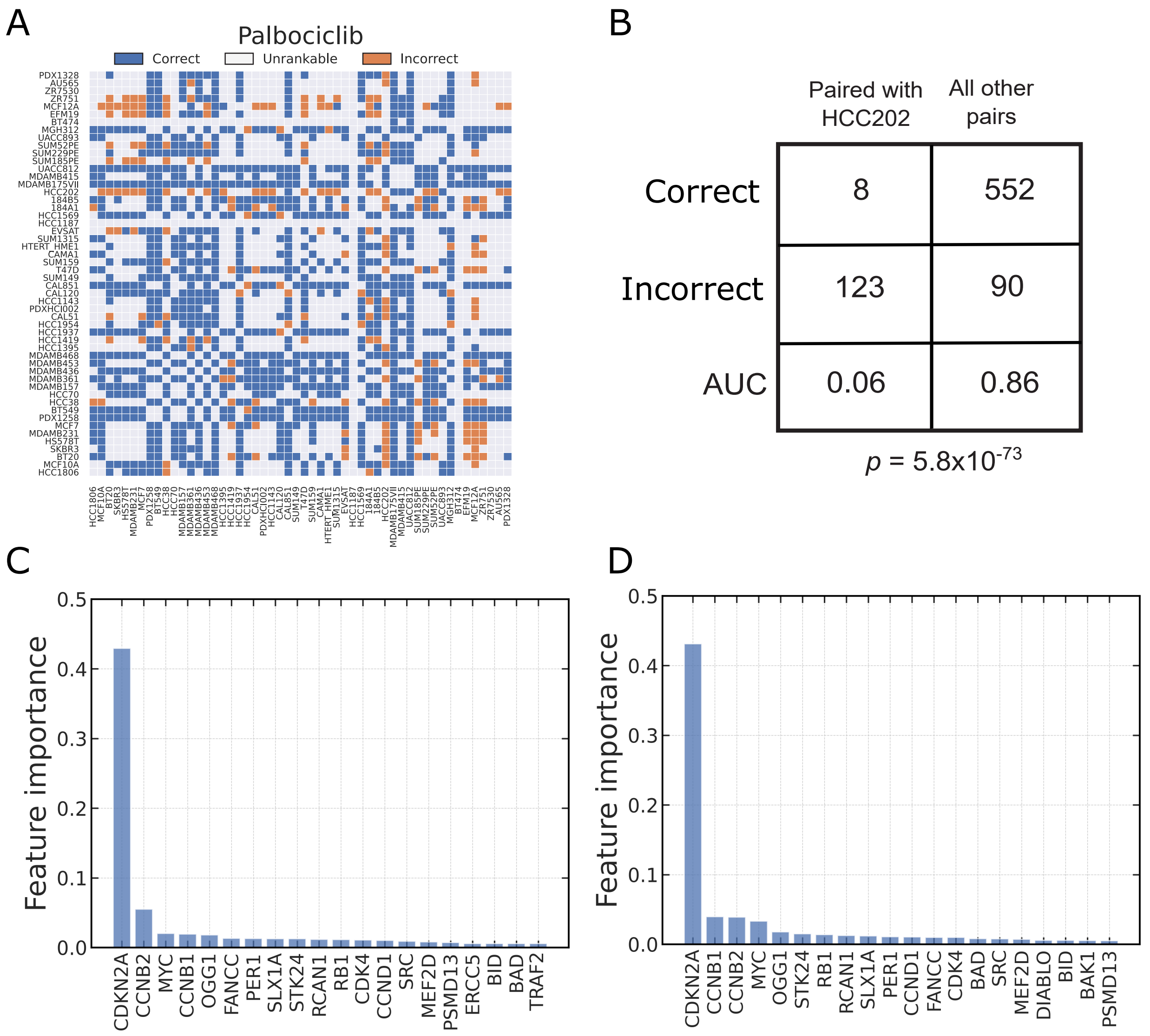


**Figure S4. Outlier detection for palbociclib.** The interpretation of all panels is analogous to Figure 3.

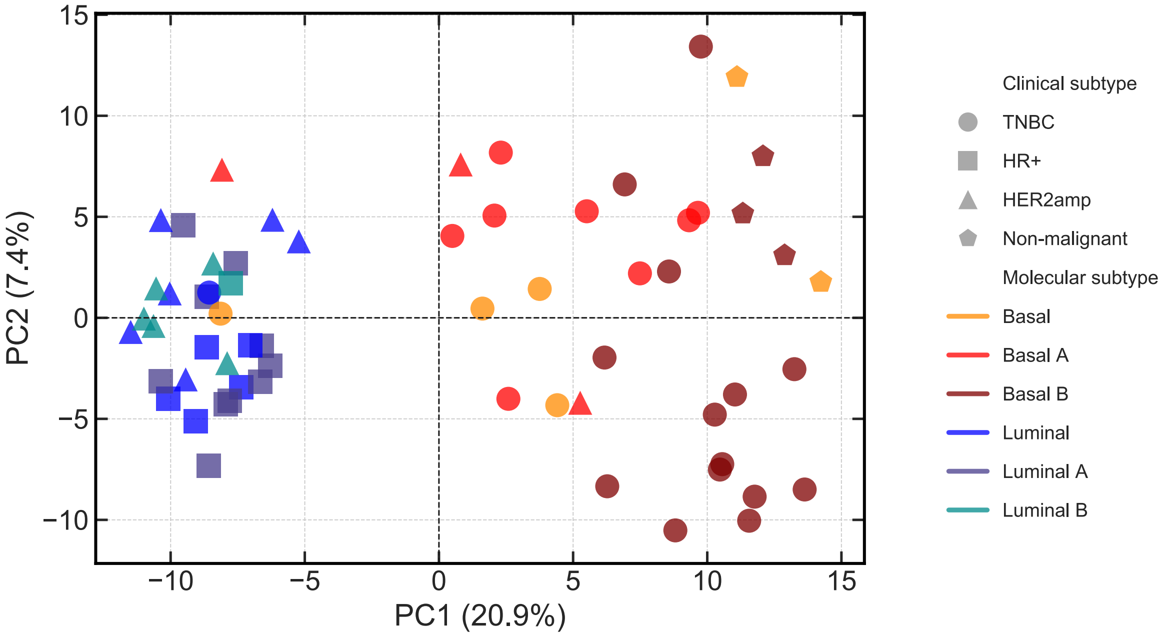


**Figure S5. Principal components analysis of baseline RNAseq expression.** The points represent individual breast cancer cell lines, shaped according to clinical subtype and colored by molecular subtype.

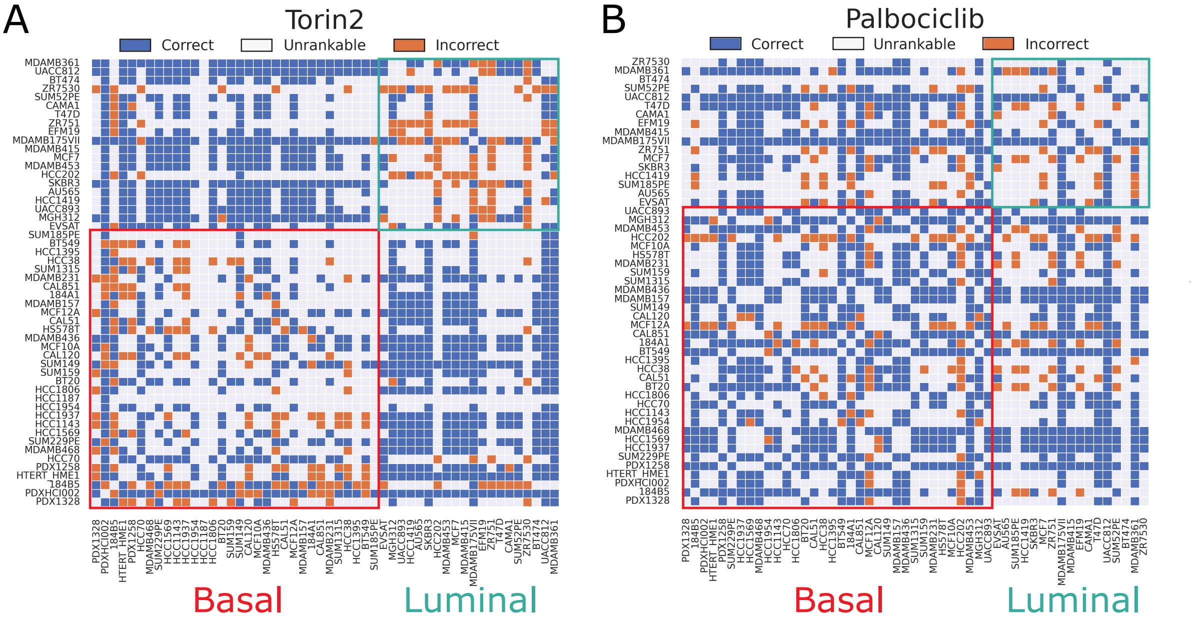


**Figure S6. Effect of subtype in model prediction for torin2 and palbociclib.**

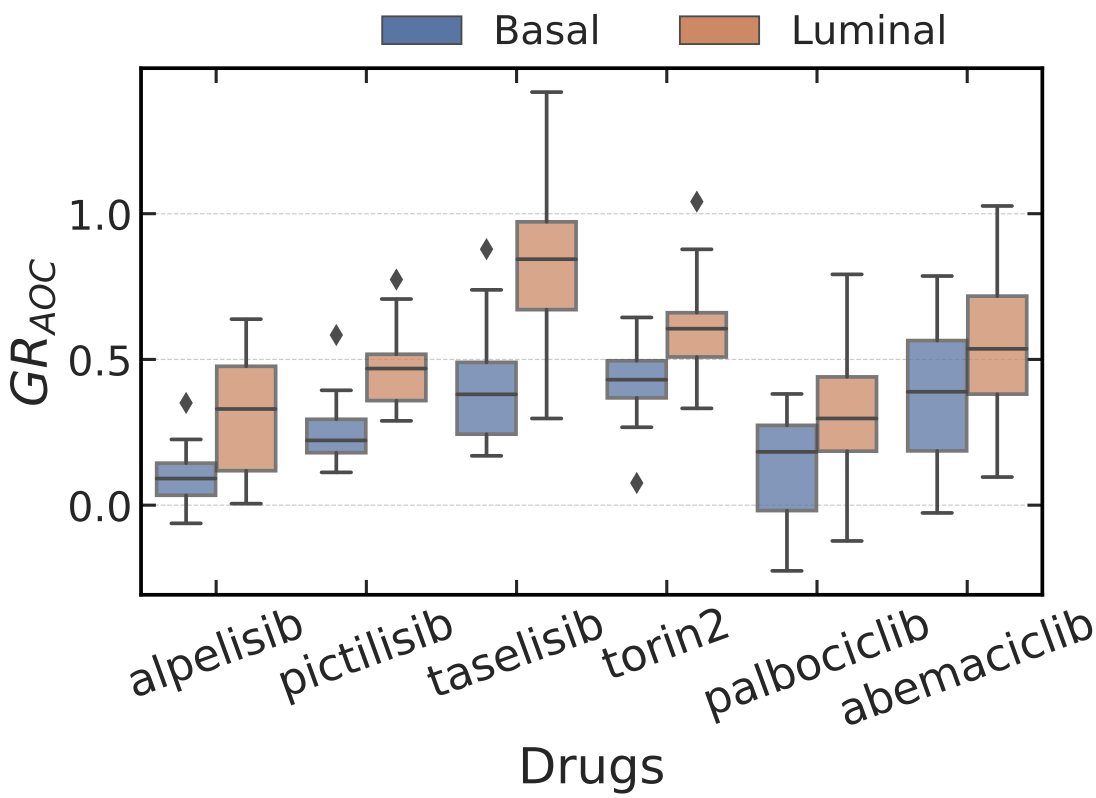


**Figure S7. The distribution of $GR_{AOC}$ for selected drugs**. The values are plotted separately for basal (blue) and luminal (orange) cell lines.

## Moderate vs mild

**Mild-centric**

| | AOD confounded | AOD matched |
|---|---|---|
| ✓ | 59 | 46 |
| ✗ | 29 | 42 |
| AUC | 0.67 | 0.52 |

$p$ = 0.03

**Moderate-centric**

| | AOD confounded | AOD matched |
|---|---|---|
| ✓ | 133 | 138 |
| ✗ | 53 | 48 |
| AUC | 0.71 | 0.74 |

$p$ = 0.76

## Moderate vs severe

**Moderate-centric**

| | AOD confounded | AOD matched |
|---|---|---|
| ✓ | 162 | 168 |
| ✗ | 40 | 34 |
| AUC | 0.80 | 0.83 |

$p$ = 0.81

**Severe-centric**

| | AOD confounded | AOD matched |
|---|---|---|
| ✓ | 51 | 60 |
| ✗ | 12 | 3 |
| AUC | 0.81 | 0.95 |

$p$ = 1.0

**Figure S8. 2x2 contingency tables showing the performance of logistic regression models trained to distinguish between mild, moderate and severe stages of Alzheimer's Disease in the presence of Age of Death (AOD) as a confounder.**

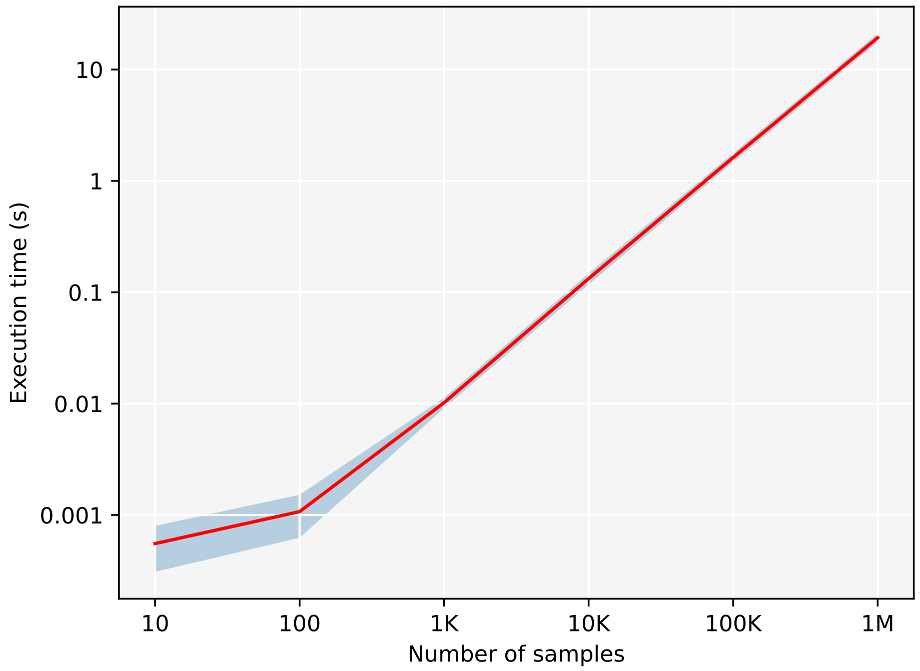


**Figure S9. Execution time of the $O(n \log n)$ paired evaluation implementation as a function of dataset size.** Shown are statistics collected over 30 runs of the method on randomly-generated sets of scores and labels. The shaded areas (blue) are one standard deviation away from the mean (red). All measurements were made in a standard Gitpod execution environment (4 vCPU, 8GB RAM) using the Python time() function.

**Table S1:** The total number of rankable pairs used to evaluate predictors of drug sensitivity from mRNA expression.

| Agent | Number of pairs | Agent | Number of pairs |
|---|---|---|---|
| Paclitaxel | 443 | BSJ-01-175 | 616 |
| Doxorubicin | 469 | BSJ-03-123 | 304 |
| Taselisib_GDC0032 | 714 | BSJ-03-124 | 435 |
| Pictilisib_GDC0941 | 358 | BVD523 | 138 |
| Torin2 | 389 | CFI-400945 | 553 |
| Vorinostat | 112 | E17 | 702 |
| Ipatasertib_GDC0068 | 333 | FMF-03-145-1 | 705 |
| Everolimus | 535 | FMF-03-146-1 | 42 |
| Tivantinib_ARQ197 | 125 | FMF-04-107-2 | 787 |
| Cabozantinib | 66 | FMF-04-112-1 | 7 |
| Saracatinib_AZD0530 | 296 | Flavopiridol | 99 |
| Dasatinib | 570 | GSK2334470 | 366 |
| Palbociclib_PD0332991 | 428 | LEE011_Ribociclib | 524 |
| Dinaciclib_SCH727965 | 224 | LY2606368 | 747 |
| AZD7762 | 592 | LY3023414 | 721 |
| Olaparib_AZD2281 | 50 | MFH-2-90 | 837 |
| Alpelisib_BYL719 | 367 | Pin1-3 | 50 |
| A-1210477 | 7 | R0-3306 | 125 |
| Buparlisib_NVP-BKM120 | 188 | Rucaparib | 198 |
| INK128_MLN0128 | 526 | SHP099 | 61 |
| PF-4708671 | 65 | SY-1365 | 735 |
| Neratinib_HKI272 | 588 | THZ-P1-2 | 356 |
| Cediranib_AZD2171 | 220 | THZ-P1-2R | 37 |
| Ceritinib_LDK378 | 205 | THZ1 | 399 |
| Trametinib_GSK1120212 | 460 | THZ531 | 352 |
| Luminespib_NVP-AUY922 | 322 | YKL-5-124 | 570 |
| Abemaciclib_LY2835219 | 559 | ZZ1-33B | 852 |
| Volasertib_BI6727 | 363 | senexin b | 150 |
| ABT-737 | 131 | | |
| TGX221 | 190 | | |
| AZD1775 | 668 | | |
| AZD2014 | 693 | | |
| AZD5363 | 531 | | |
| AZD6738 | 294 | | |
| BJP-6-5-3 | 0 | | |
| BMS-265246 | 689 | | |
